# Analyzing Large Gene Expression and Methylation Data Profiles Using StatBicRM: Statistical Biclustering-Based Rule Mining

**DOI:** 10.1371/journal.pone.0119448

**Published:** 2015-04-01

**Authors:** Ujjwal Maulik, Saurav Mallik, Anirban Mukhopadhyay, Sanghamitra Bandyopadhyay

**Affiliations:** 1 Department of Computer Science and Engineering, Jadavpur University, Kolkata, West Bengal, India; 2 Machine Intelligence Unit, Indian Statistical Institute, Kolkata, West Bengal, India; 3 Department of Computer Science and Engineering, University of Kalyani, Kalyani, West Bengal, India; Cleveland Clinic Lerner Research Institute, UNITED STATES

## Abstract

Microarray and beadchip are two most efficient techniques for measuring gene expression and methylation data in bioinformatics. Biclustering deals with the simultaneous clustering of genes and samples. In this article, we propose a computational rule mining framework, *StatBicRM* (i.e., statistical biclustering-based rule mining) to identify special type of rules and potential biomarkers using integrated approaches of statistical and binary inclusion-maximal biclustering techniques from the biological datasets. At first, a novel statistical strategy has been utilized to eliminate the insignificant/low-significant/redundant genes in such way that significance level must satisfy the data distribution property (viz., either normal distribution or non-normal distribution). The data is then discretized and post-discretized, consecutively. Thereafter, the biclustering technique is applied to identify maximal frequent closed homogeneous itemsets. Corresponding special type of rules are then extracted from the selected itemsets. Our proposed rule mining method performs better than the other rule mining algorithms as it generates maximal frequent closed homogeneous itemsets instead of frequent itemsets. Thus, it saves elapsed time, and can work on big dataset. Pathway and Gene Ontology analyses are conducted on the genes of the evolved rules using David database. Frequency analysis of the genes appearing in the evolved rules is performed to determine potential biomarkers. Furthermore, we also classify the data to know how much the evolved rules are able to describe accurately the remaining test (unknown) data. Subsequently, we also compare the average classification accuracy, and other related factors with other rule-based classifiers. Statistical significance tests are also performed for verifying the statistical relevance of the comparative results. Here, each of the other rule mining methods or rule-based classifiers is also starting with the same post-discretized data-matrix. Finally, we have also included the integrated analysis of gene expression and methylation for determining epigenetic effect (viz., effect of methylation) on gene expression level.

## Introduction

Microarray technique is a useful tool for measuring gene expression data across different experimental and control samples. Similarly, beadchip is another efficient technique for generating genome-wide DNA methylation profiling in infinium II platform. DNA methylation is an important epigenetic factor that refers to the addition of a methyl group (-CH3) to position 5 of the cytosine pyrimidine ring or the number 6 nitrogen of the adenine purine ring in genomic DNA. It modifies, in general decreases, the expression levels of genes. Both the expression and methylation data matrix [1], [2], [3], [4] are initially organized in such a way that rows and columns indicate genes and samples (conditions), respectively. Statistical analysis [5], [6], [7] is an important tool to identify differential expression/methylation (i.e., *DE*/*DM*) genes across different types of samples.

Association rule mining (ARM) [8], [9] is another useful tool for determining interesting (expression/methylation) relationships among items (genes) under different conditions (samples). In this article, we propose a computational rule mining framework, *StatBicRM* (i.e., statistical biclustering-based rule mining) to identify special rules of genes and potential biomarkers from the large gene expression and/or methylation data by integrating a novel statistical technique and binary inclusion-maximal biclustering technique, consecutively.

In traditional association rule mining algorithms, huge number of rules is coming out as result. Thus, it is difficult to run them on medium or large sized dataset in which the number of genes is approximately 250 or more. To solve the problem, in our proposed method, we have utilized the binary inclusion-maximal biclustering (i.e., BiMax) technique [10] for mining non-redundant significant itemsets and corresponding special rules. But, the biclustering technique can work on such dataset whose the number of genes is less than equal to 10,000 approximately. If the number is greater than 10,000, it fails to work. Thus, for such large dataset, we have to apply a statistical strategy on the dataset before using the biclustering technique to eliminate the redundant/insignificant/low-significant genes in such way that significance level must rely on the data distribution property (viz., either normal distribution or non-normal distribution). Therefore, first of all, the whole data is passed through different fundamental statistical techniques (viz., removal of genes having low variance and normalization, consecutively).

Now, if there is large number of samples in a dataset, there is no need to use any normality test on the data before using any statistical test as all statistical tests perform more or less well for the large number of samples. But, if a dataset has small number of samples, then it has been observed that different statistical tests perform differently [11]. It is well-known that standard t-test, Welch’s t-test, Bayes t-test and Pearson’s correlation test are all parametric statistical tests, and Limma, significant analysis of microarrays (SAM), Wilcoxon’s ranksum test and permuted t-test are considered as non-parametric tests. Some non-parametric statistical tests (like Limma and SAM) are good performers for normally distributed data as well as non-normally distributed data in all conditions, specially for small sample sizes. But, the performance of SAM is found to be inconsistent as sometimes it produces good performance while at other times it fails to work properly for small sample sizes. The performance of permuted t-test is satisfactory in case of non-normal distributions for all types of sample sizes. But, in case of normal distributions, it works poorly especially for small sample sizes. For normally distributed data, the performance of Wilcoxon’s ranksum test is much poorer than standard t-test for small sample. On the other hand, for small sample sizes, the standard t-test produces poor performance for non-normally distributed data where its performance is better in normally distributed data. Performance of Welch’s t-test is poor for both the cases of data distributions for small number of samples. To summarize, it can be stated that in case of small number of samples, it is better to test the data distribution in advance [11]. Otherwise p-values may be misleading due to the assumption of incorrect distribution. Therefore, we have initially used a well-known normality test (i.e., Jarque-Bera test [12]) for testing the distribution pattern of each data whether the data is normally distributed or not. Depending on the patterns, the dataset is then partitioned into two sub-datasets, where one sub-dataset has all normally distributed data, and remaining one contains all non-normally distributed data. Now, it is noticed that the parametric tests perform better for normally distributed data than for non-normally distributed data on average. On the other hand, the performance of non-parametric tests is more satisfactory for non-normally distributed data than for normally distributed data on average [11]. Therefore, after testing for normality, we have run multiple parametric statistical tests (viz., t-test [11], Welch’s t-test [11], modified Bayes’ t-test by Fox and Dimmic [13], and Pearson’s correlation test (Corr) [11]) on the normally distributed data to identify differentially expressed/methylated genes and taken their intersection in order to be certain that whichever genes are identified, are truly differentially expressed/methylated. Similarly, we have applied multiple non-parametric tests (viz., Limma [11], significant analysis of microarrays (SAM) [11], Wilcoxon’s ranksum test (Wcox) [11], and permuted t-test (Perm) [11]) on non-normally distributed data to obtain differentially expressed/methylated genes, and taken their intersection in order to be certain that whichever genes are identified, are truly differentially expressed/methylated. A list is then prepared containing the resulting intersected genes from both the normally distributed dataset and the non-normally distributed dataset. These statistical methods are utilized to determine the proper significant non-redundant subset of the differentially expressed/methylated genes from the original large dataset. Thereafter, discretization and post-discretization are utilized consecutively on the subset of data for converting it into corresponding boolean matrix.

Now, our next major goal is rule mining. For this purpose, the biclustering technique is directly applied on the post-discretized data-matrix for determining maximal homogeneous biclusters of genes as maximal frequent closed homogeneous itemsets (viz., *MFCHOIs*) at a minimum support-value. Here, *MFCHOI* means the maximal biclusters that have sets of all homogeneous class-labels/samples. The rules are then extracted from the *MFCHOIs*. Each evolved rule is of special type, i.e., consequent of the rule consists of its class-label only. Therefore, each *MFCHOI* produces a single special rule. Our proposed methodology performs better than state-of-the-art rule mining algorithms as it generates maximal frequent closed homogeneous itemsets (viz., *MFCHOIs*) instead of frequent itemsets. Each of the other rule mining methods is also starting with the same post-discretized data-matrix. Another advantage of it that as these rules are classification rules, so we do not need to calculate any other rule-interestingness measure (e.g. confidence) except support. Therefore, it saves elapsed time and can work on big data in which number of genes is high. Pathway and Gene Ontology (GO) analysis are conducted on the genes of the evolved rules using David database. Furthermore, frequency analysis of the genes appearing in the evolved rules is performed to determine potential biomarkers.

Furthermore, it is also needed to know how much the evolved rules are able to describe accurately the remaining test (unknown) data. For this, we need to perform cross-validation and classification, consecutively on the data to compute average accuracy of the proposed method. Therefore, the earlier mentioned post-discretized data-matrix is divided into training and test sets using 4-fold cross-validations (CVs). Thereafter, the biclustering technique is applied on the training part of the dataset for determining *MFCHOIs* at a minimum support-value. The special rules are then extracted from the *MFCHOIs*. Here, each *MFCHOI* generates a single rule. We have also estimated 23 rule-interestingness measures [14], [15] of the evolved rules. We have also added another new measure (viz., the number of satisfiable conditions/samples of the corresponding bicluster for each evolved rule). We have estimated the rank of each rule according each of the 24 measures individually using Fractional ranking [16–18]. The final ranking of each evolved rule is calculated by average ranking on the resulting fractional rankings of the rule. All the rules are rearranged from best to worst case. We have then assigned some weight on the final list of rules in such a way that the topmost rule gets the highest weight, 2nd topper gets 2nd highest weight and so on; and also the weight-interval between any two consecutive ranked rules is same. The classification technique is applied on each test data point using a majority voting technique through weighted-sum method. A comparative performance study with existing popular rule-based classifiers is conducted based on the average classification accuracy, MCC and related factors of them. Our classification method provides better performance than the existing popular rule-based classifiers. Here, each of the other rule-based classifiers is also starting with the same post-discretized data-matrix. Statistical significance tests (viz., one-way ANOVA) [19] are also performed for verifying the statistically relevance of the comparative results.

As we have mentioned earlier that DNA methylation is one of the important epigenetic factors which can change (generally decrease) the expression levels of genes, therefore we have also performed integrative analysis of gene expression dataset and methylation dataset of combined dataset. As we know that the gene expression is inversely proportional to the methylation, so inversely correlated genes make sense to highlight the epigenetic effect (e.g., methylation) on the expression level. Therefore, we have identified these type of genes having inverse relationship between their methylation and expression levels.

The rest of the article is organized as follows. In Section Materials and Methods, literature review and our proposed methodology have been elaborated. Section Results and Discussion presents source and brief description about the real datasets, and the experimental results and discussion. Finally, Section Conclusion concludes the article.

## Materials and Methods

### Literature Review

Association rule mining (ARM) [8], [9] is one of the useful tools for determining interesting (expression/methylation) relationships among items (genes) under different conditions (samples). It can provide association rules based on frequent itemsets. A rule (R) can be described as *A* ⇒ *C*, where *A*, *C* ⊆ *IM* and *A*⋂*C* = *ϕ*. Here, *A* and *C* are called as antecedent (i.e., set of items in LHS of a rule) and consequent (i.e., set of items in RHS of a rule), respectively. The support of the itemset (*IM*) is defined as number of transactions in which all items of it appear together. *IM* is frequent when its support is greater than any threshold value (i.e., minimum support). The confidence of the rule is defined as ratio of support of *IM* to the support of *A*. Frequent closed itemset (FCI) is a condensed form of frequent itemsets. FCI is used to avoid redundancy.

In past decades, traditional Apriori algorithm [20] was most fundamental association rule mining technique. Apriori uses a bottom-up technique in which frequent subsets are extended one item at a time for determining each candidate itemset. Groups of the candidate itemsets are then tested in the data. The method terminates if no further successful extension is found. The result of Apriori is the sets of rules which determine the occurrence of items in the dataset. Apriori follows breadth-first search for counting the candidate itemsets. Apriori generates candidate itemsets having length *k* from the itemsets having length *k* − 1. It discards infrequent candidate itemsets. The set of candidate itemsets have all frequent itemsets. After extracting all frequent itemsets, corresponding set of rules is mined from each frequent itemset. As Apriori generates only frequent itemsets, thus huge number of rules are produced from the itemsets. Therefore, Apriori can not run on medium or large size of data. It can hardly work up to 100 genes, approximately. But, if there is more than 100 genes, then it either takes a long time or fails to run.

After further investigations, different shortcomings have been identified in the traditional Apriori, like production of high number of frequent itemsets, high running time, problem of multiple-scan of the dataset etc. Many other ARM techniques have been proposed (e.g., AprioriTid [21], Eclat [22], Tao et al. [23], H-mine [24] etc.) to reduce these shortcomings. But, for medium or large sized dataset (i.e., whose the number of genes is greater than 250 approximately), either the methods fails to work on the dataset or they take a long time (viz., approximately 5 hours or more).

For solving the above limitation, in this article, we have used the BiMax biclustering technique [10] for extracting maximal frequent closed homogeneous itemsets (*MFCHOIs*) and corresponding special rules. It is a method for identifying groups of all-1 biclusters from a boolean data matrix under certain conditions. The aim of the biclustering is to discover groups of genes (i.e., all-1 biclusters) having similar behaviour under a subset of conditions (samples). The biclustering technique extracts the maximal frequent closed homogeneous itemsets (viz., MFCHOIs) which are proper subsets of frequent itemsets (FIs); i.e., *MFCHOI* ⊂ *FI*. Thus, Our proposed method produces much less number of significant non-redundant itemsets than the other rule mining algorithms. But, the biclustering technique can work on the dataset in which the number of genes is less than equal to 10,000 approximately. If the number is greater than 10,000, it can not work on the dataset. Therefore, for the large dataset, we need to utilize some statistical strategy on the dataset before applying the biclustering technique for eliminating the redundant/insignificant/low-significant genes in such a way that significance level must satisfy the data distribution property (viz., either normal distribution or non-normal distribution). Hence, here, we have proposed a computational rule mining framework, *StatBicRM* for producing special rules of genes, and potential biomarkers from the large gene expression and/or methylation dataset by integrating a novel statistical technique and the biclustering technique, consecutively.

### Proposed Method

**Fig 1:**
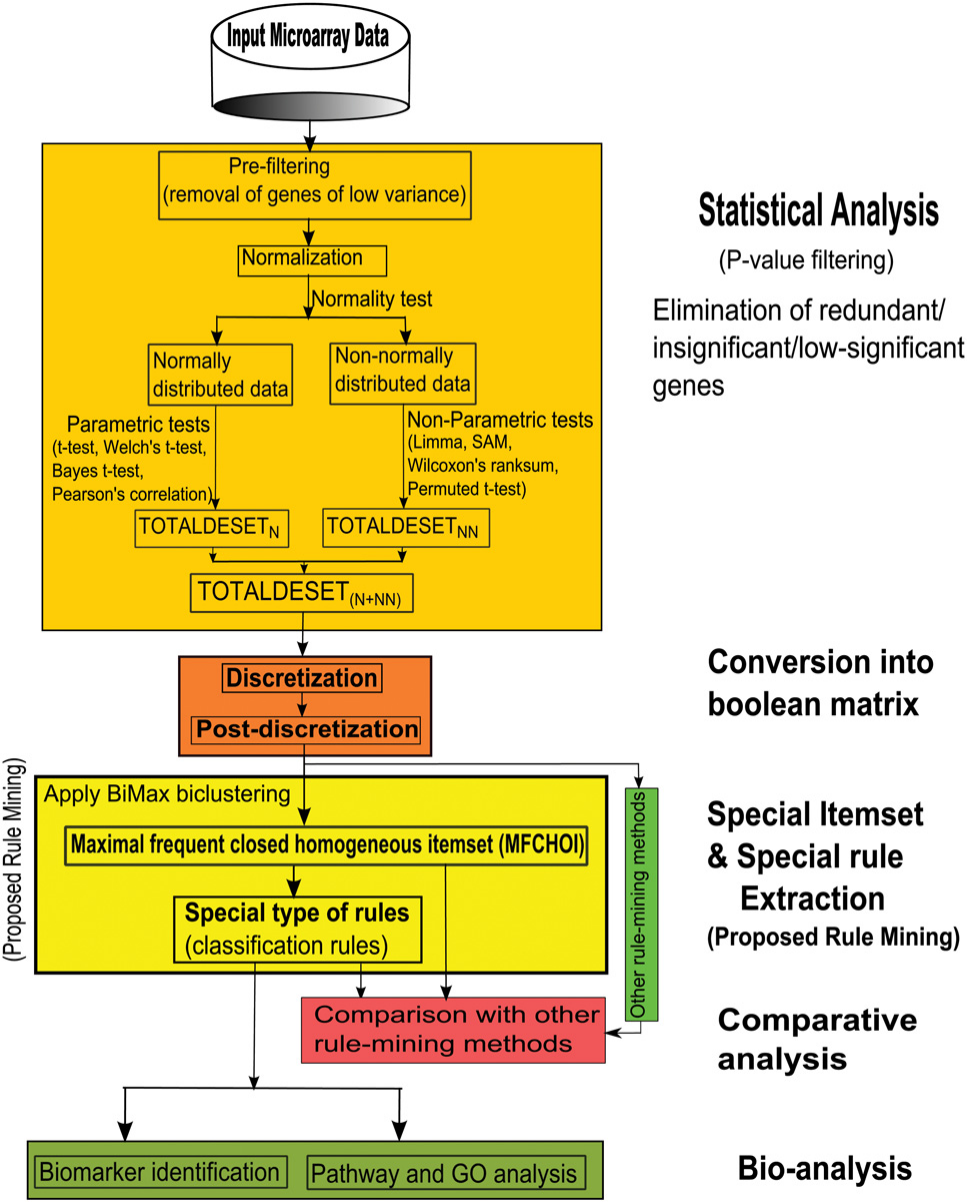
Flowchart of the proposed methodology (*StatBicRM*) for the rule mining. Here, the terms *TOTALDESET*_*N*_, *TOTALDESET*_*NN*_, *TOTALDESET*_*N*+*NN*_ are described in last paragraph of subsection *“Identification of differentially expressed/methylated genes using Statistical tests”*. For methylation dataset, the above terms are replaced by *TOTALDMSET*_*N*_, *TOTALDMSET*_*NN*_, *TOTALDMSET*_*N*+*NN*_, respectively.

**Fig 2:**
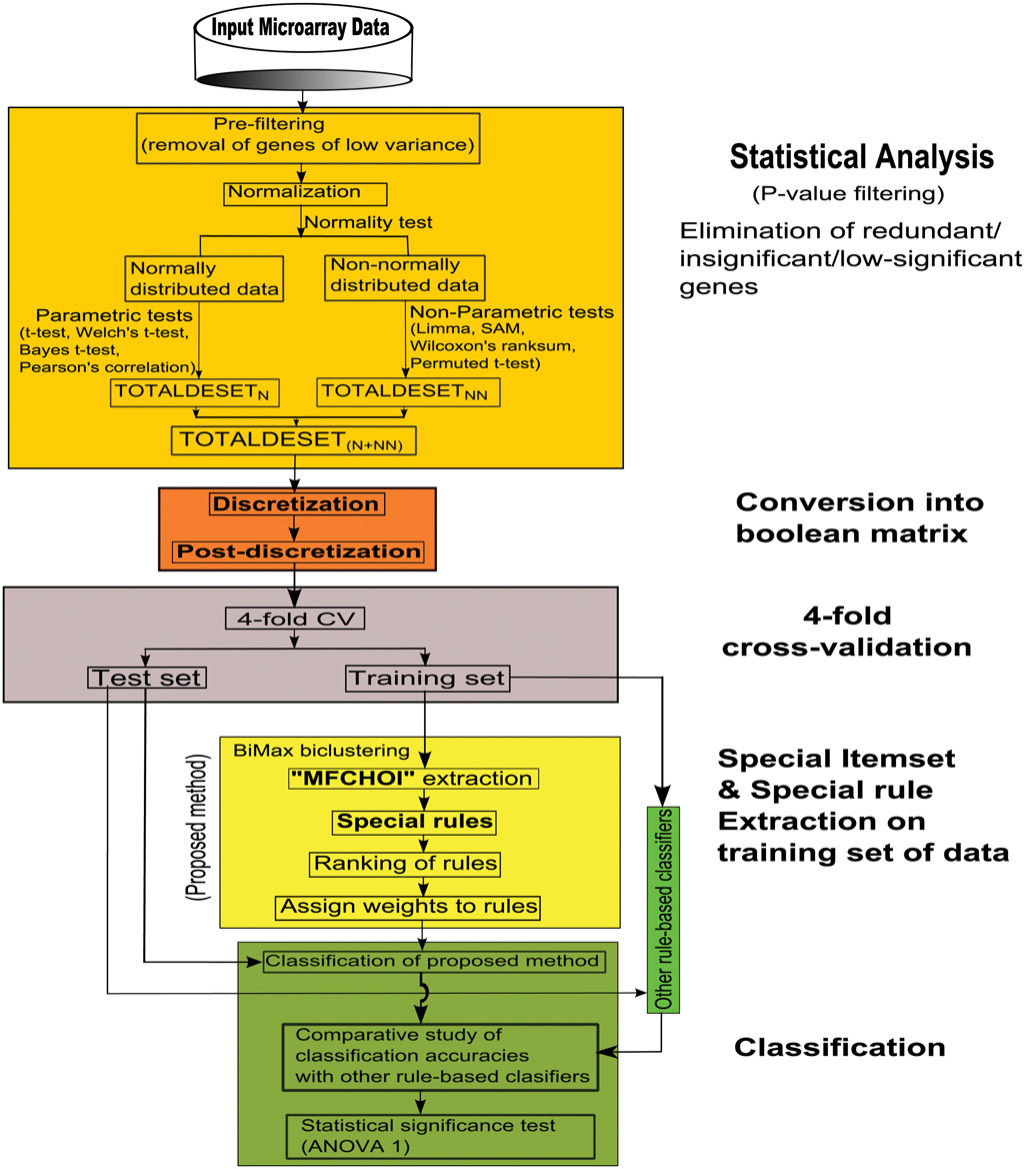
Flowchart of the proposed methodology (*StatBicRM*) for the classification. Here, the terms *TOTALDESET*_*N*_, *TOTALDESET*_*NN*_, *TOTALDESET*_*N*+*NN*_ are described in last paragraph of subsection. For methylation dataset, the above terms are replaced by *TOTALDMSET*_*N*_, *TOTALDMSET*_*NN*_, *TOTALDMSET*_*N*+*NN*_, respectively.

Our proposed technique, *StatBicRM* is basically a computational framework for rule mining where integrated approach of statistical and binary inclusion-maximal biclustering techniques are utilized in gene expression or methylation dataset (see Fig. 1). Besides this, we have also performed classification using the proposed method to know how much the evolved rules are able to describe accurately the remaining test (unknown) data (see Fig. 2).

The steps of *StatBicRM* is described briefly in the following steps:

#### Identification of differentially expressed/methylated genes using Statistical tests

Our proposed method basically depends on statistical analysis. As we know that in case of big gene expression/methylation dataset, there may exist 10,000 or more genes. Among them, most of the genes are non-differentially expressed/methylated (i.e., *nDE*/*nDM*), and only some of them are differentially expressed/methylated (i.e., *DE*/*DM*). When a rule is generated, then these two types of genes may occur together in the rule. According to biological scenario, *DE*/*DM* genes can only make sense in a rule relating to specific disease, where the other type of genes is irrelevant to the disease. Therefore, we have initially used a novel statistical strategy on the dataset to identify the set of statistically significant non-redundant *DE*/*DM* genes in such way that significance level must rely on the data distribution property (viz., either normal distribution or non-normal distribution). For doing this, at first, the genes which have low variance are eliminated from the gene expression/methylation dataset. Thereafter, we have used zero-mean normalization on the data of these genes to adjust the values measured on different scales to a common scale. The zero-mean normalization can be stated as:

$$\begin{array}{c} v_{ij}^{\prime }=\frac{v_{ij}-\mu }{\sigma }, \end{array}$$ (1)

where *μ* and *σ* denote mean and standard deviation of the expression/methylation data of a gene *i* before normalization respectively; and *v*_*ij*_ and $v_{ij}^{\prime }$ refer to the value of *i*-th gene at *j*-th condition before and after normalization, respectively.

It is well known that the parametric statistical tests [25] are appropriate for normally distributed data, and non-parametric statistical tests [25] are appropriate for non-normally distributed data, respectively. Therefore, Jarque-Bera normality test [12], [26] is utilized on the normalized data to determine the pattern of distribution of the data whether it is normally distributed or non-normally distributed. The Jarque-Bera normality test is defined as follows:

$$\begin{array}{c} JB=\frac{d}{6}\left(S^{2}+\frac{1}{4}{\left(K-3\right)}^{2}\right), \end{array}$$ (2)

where *d* denotes the degree of freedom, *S* is the skewness of the sample, and *K* refers to the kurtosis of the sample. Hence, depending on the resulting distribution patterns, the whole normalized dataset is partitioned into two sub-datasets, where one sub-dataset has all normally distributed data, and remaining one contains all non-normally distributed data.

Thereafter, we have applied four parametric statistical tests (viz., t-test [11], Welch’s t-test [11], modified Bayes’ t-test by Fox and Dimmic [13] in 2006 and Pearson’s correlation test (Corr) [11]) on the normally distributed data to obtain differentially expressed genes for the normally distributed sub-dataset. Similarly, four non-parametric tests (viz., Limma [11], Significant analysis of microarrays (SAM) [11], Wilcoxon ranksum test (Wcox) [11] and permute t-test (Perm) [11]) are applied on the non-normally distributed data to obtain differentially expressed genes for the non-normally distributed sub-dataset.

Before further proceeding, we have shortly discussed in the followings about some of the statistical tests mentioned above.

The “2-sample t-test” makes comparison between means of the two groups with the variation in the data. From the test statistic, we compute a measure (i.e., p-value). The p-value indicates the probability of observing a t-value as large or larger than the actually observed t-value where the null hypothesis is given true. By convention, if the p-value of a gene (item) is less than 5%, then the gene is statistically called as differentially expressed/methylated gene. Now, suppose, for each gene *g*, group 1: *n*_1_ treated samples, with mean ${\bar{x}}_{1g}$ and standard deviation *s*_1*g*_; and group 2: *n*_1_ controlled samples, with mean ${\bar{x}}_{2g}$ and standard deviation *s*_2*g*_.

$$\begin{array}{c} t=\frac{\left({\bar{x}}_{1g}-{\bar{x}}_{2g}\right)}{se_{g}}. \end{array}$$ (3)

Here, *se*_*g*_ denotes the standard error of the groups’ mean, thus,

$$\begin{array}{c} se_{g}=sPooled*\sqrt{\frac{1}{n_{1}}+\frac{1}{n_{2}}}, \end{array}$$ (4)

where *sPooled* is the pooled estimate of the population standard deviation; i.e.,

$$\begin{array}{c} sPooled=\sqrt{\frac{\left(n_{1}-1\right)*s_{1g}^{2}+\left(n_{2}-1\right)*s_{2g}^{2}}{df}}. \end{array}$$ (5)

Here, *df* is degree of freedom of the test. It is stated as *df* = (*n*_1_ + *n*_2_ − 2). This strategy is used assuming that variance of two groups are equal.

For Welch’s t-test, the variance of two groups are checked whether they are equal to each other or not. If equal, then use earlier mentioned t-statistic in Equation 3, otherwise use the following t-statistic:

$$\begin{array}{c} t=\frac{\left({\bar{x}}_{1g}-{\bar{x}}_{2g}\right)}{\sqrt{\frac{s_{1g}^{2}}{n_{1}}+\frac{s_{2g}^{2}}{n_{2}}}}. \end{array}$$ (6)

Here we use unpooled estimates of the population standard deviations.

Pearson’s correlation coefficient (commonly denoted as *ρ*) between two variables is described as the covariance of the two variables divided by the product of their standard deviations, i.e.,

$$\begin{array}{c} \rho =\frac{cov(x,y)}{s_{x}s_{y}}, \end{array}$$ (7)

where

$$\begin{array}{c} cov\left(x,y\right)=\sum_{i=1}^{n1}\left(x_{i}-\bar{x}\right)\left(y_{i}-\bar{y}\right), \end{array}$$ (8)

where samplesize for the two groups are *n*1 and *n*2, respectively (here, *n*1 = *n*2). This test can predict whether two variables are related or not.

The moderated t-statistic in Limma [27] can be demonstrated as:

$$\begin{array}{c} {\tilde{t}}_{g}=\frac{1}{\sqrt{\frac{1}{n_{1}}+\frac{1}{n_{2}}}}\frac{{\hat{\beta }}_{g}}{{\tilde{s}}_{g}}, \end{array}$$ (9)

where samplesize *n* = *n*_1_ + *n*_2_, ${\hat{\beta }}_{g}$ and ${\tilde{s}}_{g}^{2}$ denote the contrast estimator and posterior sample variance for the gene *g* respectively. The statistic for calculating contrast estimator for gene *g* is:

$$\begin{array}{c} {\hat{\beta }}_{g}|\sigma_{g}^{2}∼N\left(\beta_{g},\sigma_{g}^{2}\right), \end{array}$$ (10)

where, *N* is normal distribution, and the statistic for estimating posterior sample variance for the gene *g* is:

$$\begin{array}{c} {\tilde{s}}_{g}^{2}=\frac{d_{0}s_{0}^{2}+d_{g}s_{g}^{2}}{d_{0}+d_{g}}. \end{array}$$ (11)

Where, *d*_0_ (< ∞) and $s_{0}^{2}$ refer to the prior degrees of freedom and variance respectively, and *d*_*g*_ (> 0) and $s_{g}^{2}$ denote the experimental degrees of freedom and the sample variance of a particular gene *g*, respectively.

SAM chooses to add a small positive constant *s*_0_ (stated as “fudge factor”) to solve small variance problem. The SAM statistic by Tusher *et al*.(2001) is:

$$\begin{array}{c} t_{sam}=\frac{\left({\bar{x}}_{1g}-{\bar{x}}_{2g}\right)}{se_{g}+s_{0}}, \end{array}$$ (12)

where *se*_*g*_ is the standard error of the groups’ mean (see Equation 4). Here, *sPooled* is the pooled estimate of the population standard deviation (see Equation 5). Here, *df* is degree of freedom of the test. It is stated as *df* = (*n*_1_ + *n*_2_ − 2).

In Wilcoxon ranksum test, a list of ranks of the gene expression values for each gene is prepared in ascending for each group, and then tests for equality of means of the two ranked samples. The z-statistic of the test is:

$$\begin{array}{c} z=\frac{\left(|T-mean_{w1}|-0.5\right)}{\sqrt{var_{w1}}}, \end{array}$$ (13)

where

$$\begin{array}{c} T=min\left(\sum ranks_{group1},\sum ranks_{group2}\right), \end{array}$$ (14)

$$\begin{array}{c} mean_{w1}=n_{1}*\left(n_{1}+n_{2}+1\right)/2, \end{array}$$ (15)

and

$$\begin{array}{c} var_{w1}=n_{2}*mean_{w1}/6. \end{array}$$ (16)

A permuted t-test is a kind of t-test in which an rearrangement is conducted in the labels on the observed data-points of each gene (item).

However, as stated earlier that the four parametric statistical tests are applied on the normally distributed dataset, thus different number of up-regulated and down-regulated genes are coming out from the different parametric tests. Thereafter, we have performed intersection of the up-regulated genes to identify *set of common up-regulated genes* (denoted by *UPDESET*_*N*_) for the *normally distributed sub-dataset*. Similarly, we have got *set of common down-regulated genes* (denoted by *DOWNDESET*_*N*_). We have then made a list (denoted by *TOTALDESET*_*N*_) containing all the common up-regulated genes and all the common down-regulated genes; i.e., *TOTALDESET*_*N*_ = *UPDESET*_*N*_ + *DOWNDESET*_*N*_. Similarly, as stated earlier that the four non-parametric statistical tests are applied on the non-normally distributed dataset, thus different number of up-regulated and down-regulated genes are coming out from the different non-parametric tests. Then, we have made intersection of the up-regulated genes to identify *set of common up-regulated genes* (denoted by *UPDESET*_*NN*_) for the *non-normally distributed sub-dataset*. Similarly, we have got *set of common down-regulated genes* (denoted by *DOWNDESET*_*NN*_). We have then made another list (denoted by *TOTALDESET*_*NN*_) containing all the common up-regulated genes and all the common down-regulated genes; i.e., *TOTALDESET*_*NN*_ = *UPDESET*_*NN*_ + *DOWNDESET*_*NN*_.

Finally, we have produced a final list (denoted by *TOTALDESET*_(*N*+*NN*)_) containing all the common up-regulated and down-regulated genes from the normally distributed and non-normally distributed datasets; i.e., *TOTALDESET*_(*N*+*NN*)_ = *TOTALDESET*_*N*_ + *TOTALDESET*_*NN*_. Hence, the final list of genes (i.e., *TOTALDESET*_(*N*+*NN*)_) are utilized in the next step. Similar steps are performed to obtain *HYPERDMSET*_*N*_, *HYPODMSET*_*N*_, *TOTALDMSET*_*N*_, *HYPERDMSET*_*NN*_, *HYPODMSET*_*NN*_, *TOTALDMSET*_*NN*_ and *TOTALDMSET*_(*N*+*NN*)_ instead of *UPDESET*_*N*_, *DOWNDESET*_*N*_, *TOTALDESET*_*N*_, *UPDESET*_*NN*_, *DOWNDESET*_*NN*_, *TOTALDESET*_*NN*_, *TOTALDESET*_(*N*+*NN*)_ respectively.

#### Discretization and Post-discretization

**Fig 3:**
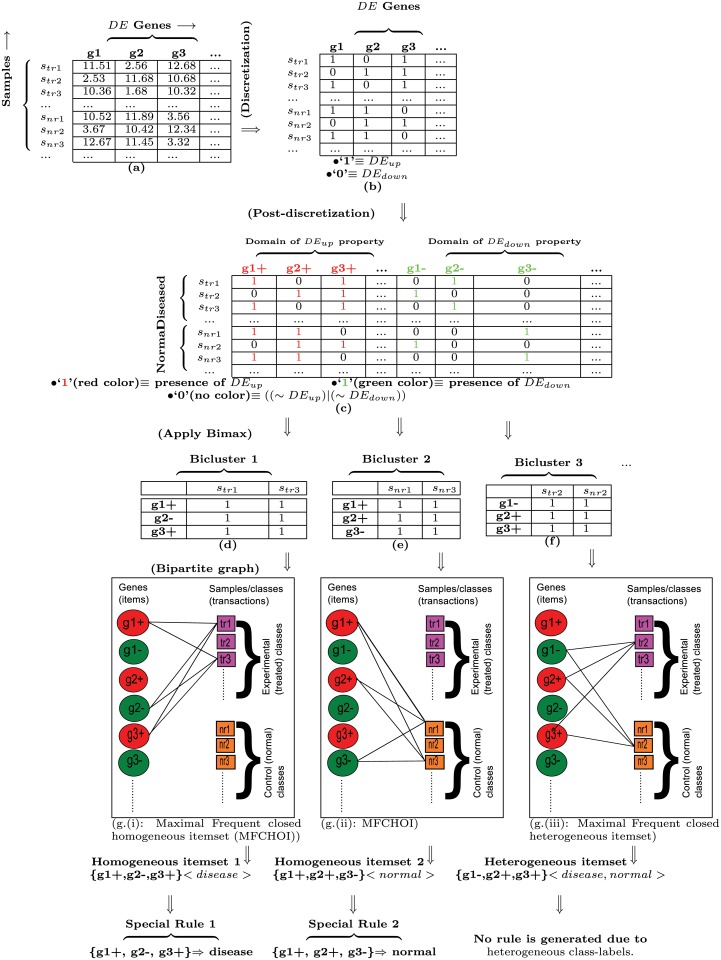
An example of generating special rules from data matrix of the differentially expressed genes. Here, up-regulation (i.e., ‘+’) and down-regulation (‘-’) are denoted by ‘1’ and ‘0’ in (b), and red and green colors in (c), respectively. Here, *s*_*tr*_ and *s*_*nr*_ denote experimental/diseased/treated and control/normal samples respectively.

Suppose, the data matrix of the *resulting list of genes*, *TOTALDESET*_(*N*+*NN*)_ is denoted by *I*. Now, first of all, *I* whose rows denote genes and columns denote samples, is transposed. Suppose, *PIT* is the transposed matrix. As the *PIT* matrix is already normalized by zero-mean normalization, therefore the following step is utilized for binary discretization of the matrix:

$$\begin{array}{c} IT=\left\{\begin{array}{cc} 1, & \text{if}\;PIT_{ij}>0,\\ 0, & \text{if}\;PIT_{ij}<0; \end{array}\right. \end{array}$$ (17)

where *PITij* denotes the expression/methylation value of *i*-th row and *j*-th column (1 ≤ *i* ≤ *m*, 1 ≤ *j* ≤ *n*), *m* and *n* are number of rows (samples) and number of columns (genes) of *PIT*_*ij*_ matrix, respectively, and *IT* is the resulting discretized matrix. Now, let us assume that in the discretized boolean matrix, a up-regulated gene and a down-regulated gene are denoted by *DE*_*up*_ and *DE*_*down*_, respectively. In the matrix *IT*, ‘1’ and ‘0’ refer to presence of up-regulated gene (*DE*_*up*_), and presence of down-regulated gene (*DE*_*down*_), respectively (see part (b) of Fig. 3). After discretization, we will apply Bimax biclustering for finding all-1 biclusters. As the Bimax biclustering rectifies only ‘1’, not ‘0’, thus we need to do post-discretization in such way where ‘1’ will represent both *DE*_*up*_ and *DE*_*down*_ properties. Therefore, after discretization, number of columns is doubled where the first half is a domain for *DE*_*up*_ property, and remaining half is another domain for *DE*_*down*_ property (see part (c) of Fig. 3). E.g., the column denoted by *g*1 in part (b) of Fig. 3 is divided into the two columns denoted by *g*1+ and *g*1− in part (c) of Fig. 3, where for the *g*1+ column, ‘1’ denotes presence of up-regulated gene (*DE*_*up*_) and ‘0’ denotes absence of up-regulated gene (∼ *DE*_*up*_), and for the *g*1− column, ‘1’ denotes presence of down-regulated gene (*DE*_*down*_) and ‘0’ denotes absence of down-regulated gene (∼ *DE*_*down*_). Hence, for methylation data, *DM*_*hyper*_ and *DM*_*hypo*_ are used instead of *DE*_*up*_ and *DE*_*down*_, respectively. Note that in this paper, ‘+’ and ‘-’ denote up-regulation/hyper-methylation and down-regulation/hypo-methylation, respectively.

#### Dividing whole data into training and test sets

Let us assume that the post-discretized matrix is denoted by *ITb*. For classification of the matrix *ITb*, we have applied 4-fold cross-validations (CVs) on the matrix to divide it into test and training data, where one-fold of *ITb* will be used as test set and remaining three fold will be considered as training set. This procedure will be repeated for four times as it is 4-fold CV.

#### Finding maximal biclusters and extracting special rules

We have transposed the training boolean dataset, and applied Bimax biclustering to identify maximal frequent closed homogeneous itemsets (MFCHOIs). Before further proceeding, the fundamental method of BiMax biclustering is discussed in short.

Suppose, a boolean matrix *e* has size of *n* × *m*, where *n* is number of genes and *m* is the number of samples. A cell *e*_*ij*_ is 1 if gene *i* expresses differentially in the sample/condition *j* and otherwise, *e*_*ij*_ is 0. A bicluster (*G*, *S*) is a subset of genes *G* ⊆ {1, 2, …, *n*} which express differently together under a subset of samples *S* ⊆ {1, 2, …, *m*}; i.e., the pair (*G*, *S*) refers to a subset of the matrix *e* whose all elements have 1. The biclusters which are inclusion-maximal (i.e., the biclusters that are not entirely part of any other bicluster), are only interesting. The pair (*G*, *S*) ∈ 2^{1,2,…,*n*}^ × 2^{1,2,…,*m*}^ can be stated as a bicluster of the type inclusion-maximal [10] if and only if (i) *e*_*ij*_ = 1, ∀*iεG*, *jεS*, and (ii) ∄ (*G*′, *S*′) ∈ 2^{1,2,…,*n*}^ × 2^{1,2,…,*m*}^ with (a) *e*_*ij*_ = 1, ∀*i*′ ∈ *G*′, *j*′ ∈ *S*′ and (b) *G* ⊆ *G*′∧*S* ⊆ *S*′∧(*G*′, *S*′) ≠ (*G*, *S*). When there is no proper superset of an itemset have been found at the same support value, then the itemset is called closed itemset. Finding the set of frequent itemsets is totally equivalent to get a set of all-1 biclusters each having at least number of conditions/samples (i.e., satisfying minimum support).

In our experiment, for the biclustering, we have set a fixed minimum cutoff of items/genes (viz., 2), and different minimum cutoffs of sample/condition for determining itemsets at different minimum support of each rule. The BiMax biclustering can generate all maximal biclusters. The items/genes of maximal biclusters represent a (maximal) closed itemset. Thus, all extracted biclusters that are satisfying minimum support condition, produce the set of (maximal) frequent closed itemsets with their class-labels (i.e., conditions/types of samples). Thereby, we have to filter the biclusters depending on their conditions. We have selected such (maximal) biclusters which have the group of homogeneous (non-contradictory) conditions. In other words, we have to identify maximal frequent closed homogeneous itemsets (*MFCHOIs*). E.g., Bicluster 1 is a *MFCHOI* which has three genes *g*1+, *g*2− and *g*3+, and two homogeneous conditions/samples *s*_*tr*1_ and *s*_*tr*3_ (presented in part (d) of Fig. 3). Similarly, Bicluster 2 is another *MFCHOI* which has three genes *g*1+, *g*2+ and *g*3−, and two homogeneous conditions/samples *s*_*nr*1_ and *s*_*nr*3_ (presented in part (e) of Fig. 3). Hence, we have omitted the biclusters that have the group of heterogeneous (contradictory) conditions. E.g., Bicluster 3 is such type of heterogeneous (contradictory) bicluster which has three genes *g*1−, *g*2+ and *g*3+, and two heterogeneous conditions *s*_*tr*2_ and *s*_*nr*2_ (presented in part (f) of Fig. 3).

From each selected bicluster of genes, we can extract an association rule. Each resulting rule must be of special type, i.e., consequent of the rule consists of its class-label (i.e., either treated/diseased/experimental class-label or normal/control class-label) only. E.g., from the Bicluster 1 (depicted in part (d) of Fig. 3), rule id 1 (i.e., {*g*1+, *g*2−, *g*3+} ⇒ *disease*) is produced. It states that if both of gene1 and gene3 are up-regulated/hyper-methylated and gene2 is down-regulated/hypo-methylated simultaneously, then ‘disease’ occurs (see part (d) and part (g).(i) of Fig. 3). Similarly, rule id 2 (i.e., {*g*1+, *g*2+, *g*3−} ⇒ *normal*) is generated from the Bicluster 2 (see part (e) and part (g).(ii) of Fig. 3).

#### Ranking of rules

We have evaluated each evolved rule based on 24 rule-interestingness measures. Support, confidence, coverage, prevalance, sensitivity (or, recall), specificity, accuracy, lift (or, interest), leverage, added value, relative risk, Jaccard, Yules’ Q, klosgen, Laplace correction, Gini index, two-way support, linear correlation coefficient (or, *ϕ*-coefficient), cosine, least contradiction, Zhang, liverage2 (or, Piatetsky-Shapiro) and kappa [14, 15] are already included among the 24 measures (see S1 Text). The last and novel measure is the number of satisfiable conditions/samples to each evolved rule. E.g., according to part (g).(i) of Fig. 3, the value of the measure of rule id 1 (i.e., {*g*1+, *g*2−, *g*3+} ⇒ *disease*) is 2 as its corresponding bicluster (in part (d) of Fig. 3) has two conditions (*s*_*tr*1_ and *s*_*tr*3_). Similarly, according to part (g).(ii) of Fig. 3, the value of the measure of rule id 2 (i.e., {*g*1+, *g*2+, *g*3−} ⇒ *normal*) is 2. The rank of the rule is proportional to the value of the measure of it (i.e., if a rule that has higher value of the measure than other rule, then the rank of the rule will be better than the second rule).

Thereafter, the evolved rules are ranked according to each of the 24 rule-interestingness measures individually using Fractional ranking [16–18]. In the fraction ranking, items which compare equal, hold the same rank. This rank is the mean of ranking numbers that are received in ordinal ranking. E.g., suppose, a data set is {1 2 2}. Here, only two different numbers are available, so there should be two different ranks. If 2 and 2 are actually different numbers, then they should hold ranks 2 and 3, respectively. As these two numbers are same, thus we should calculate their rank by making the average of their ranks as follows: (2+3)/2 = 2.5; therefore, the fractional ranks will be: 1 2.5 2.5.

Hence, the final ranking of each rule is determined by average ranking on the resulting fractional rankings of the rule. The rules are then rearranged in ascending order (i.e., from best to worst rank).

#### Assigning weights to the rules w.r.t. their final ranking

For classification, we have to apply a majority voting technique on each test data point to identify its class-label through weighted-sum method. Thus, we firstly assign some weight on the final list of rules in such a way that the topmost rule gets the highest weight, 2nd topper gets 2nd highest weight and so on. The weight of the first ranked rule is always 1. The ranges of weight lie in between 0 and 1. The weight of each rule (denoted by *w*_*j*_, 1 ≤ *j* ≤ *p*) is estimated from a function of its final rank of the rule (denoted by *r*_*j*_) and the total number of rules (viz., *p*) as described below:

$$\begin{array}{c} w_{j}=\frac{1}{p}*\left(p-\left(r_{j}-1\right)\right). \end{array}$$ (18)

Here, the weight-interval between any two consecutive ranked rules is same. Thus, the calculated weights of the rules are normalized using zero-mean normalization (in Equation 1).

#### Majority voting and classification

**Fig 4:**
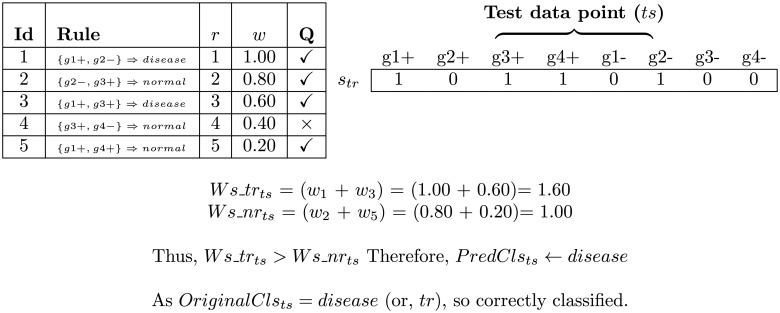
An example of classification of evolved rules by the majority voting using weighted-sum. Here, ‘r’ and ‘w’ denote rank and weight of the rule (computed by Equation 18), respectively. Tickmark/crossmark in ‘Q’ column states that test-point (*ts*) is satisfied/non-satisfied by the corresponding rule.

Consider one test data point (*ts*). For determining of the predicted class-label of it, we have applied ‘majority voting’ technique. At first, we have identified the rules whose all the items (genes) in their antecedent sides exist in *ts*. The weights of the rules (i.e., *trR*_*ts*_ number of rules) which have only the class-label ‘disease’ in their consequent sides are then summed up (viz., *Ws*_*tr*_*ts*_). Similar summation (viz., *Ws*_*nr*_*ts*_) is performed for the rules (i.e., *nrR*_*ts*_ number of rules) having only the class-label ‘normal’ in their consequent sides. The two weighted-sum are then compared, and the class-label with higher weighted-sum becomes the predicted class-label of *ts* (viz., *PredCls*_*ts*_). But, if both the weighted-sum are equal to each other, then the class-label of the top rule which satisfies *ts* (i.e., *ClsTopR*_*ts*_), becomes the predicted class-label of it. In case, if there is no such rule which satisfies that *ts*, then the class-label of the rule which satisfies maximum number of test points (i.e., *Cls*_*R*′_) becomes the predicted class-label of it. An example of majority voting technique is presented in Fig. 4. Repeat this step for other test points. This process is then repeated 4 times for 4 sub-matrices of the test data as here 4-fold CV is used. Using this technique, we have calculated true positive (TP), true negative (TN), false positive (FP), false negative (FN), sensitivity [7], specificity [7], accuracy [7] and Mathews correlation coefficient (MCC) [7] for the proposed classification. Sensitivity, specificity, accuracy and MCC are defined in the followings, respectively:

$$\begin{array}{c} sensitivity=\frac{TP}{\left(TP+FN\right)}, \end{array}$$ (19)

$$\begin{array}{c} specificity=\frac{TN}{\left(FP+TN\right)}, \end{array}$$ (20)

$$\begin{array}{c} accuracy=\frac{\left(TP+TN\right)}{\left(TP+FP+TN+FN\right)}, \end{array}$$ (21)

$$\begin{array}{c} MCC=\frac{\left(TP*TN)-(FP*FN\right)}{\sqrt{\left(TP+FP)(TP+FN)(TN+FP)(TN+FN\right)}}. \end{array}$$ (22)

we have repeated the 4-fold CV for 10 times, and then their average sensitivity, average specificity, average accuracy, and average MCC are calculated with the standard deviations based on the results of cross-validations. Thereafter, a comparative performance analysis has been conducted between our proposed method (i.e., *Prop*) and other popular rule-based classifiers (i.e., ConjunctiveRule (CJR) [28], DecisionTable (DT) [28], JRip [28], OneR [28], PART [28] and Ridor [28] implemented in Weka 3.6 software) based on their average sensitivity, average specificity, average accuracy, and average MCC. Note that the other rule-based classifiers are also started with the same post-discretized matrix (i.e., *ITb*). We have also performed significance test (viz., One-way Anova) on the accuracies of the classifiers pairwise to know the level of significance (i.e., p-value) of the test for each (pairwise) comparison.

The above steps have been described the proposed methodology for the classification.

#### Performance comparison with other rule mining algorithms

For the purpose of rule mining only, the whole post-discretized data matrix (i.e., *ITb*) is used directly as a input of the Bimax biclustering. In this case, we have not performed any cross-validation since there is no need to use classification. Hence, we have compared our proposed rule mining algorithms with the other existing popular rule mining algorithms (i.e., AprioriTid [21], Eclat [22], Tao et al. [23] and H-mine [24]). It should be noted that same input binary matrix (i.e., *ITb*) is utilized for the other rule mining algorithms.

#### Biological significance of evolved rules, and Biomarker identification

As all the real datasets are microarray/beadchip (biological) datasets, so the evolved top rules should have biological significance. The information about the relation between the genes and any disease can be determined from pathway and Gene Ontology (GO) analyses. If all the genes (except the class-label) of a rule occur together in any pathway/GO-term and if the occurrence is statistically significant (i.e., p-value is less than 0.05), then the rule becomes the (statistically) biologically significant rule. If the pathway/GO-term relates to the corresponding disease, then the rule becomes important for diagnosing the disease. Therefore, KEGG pathway and GO analyses have been performed on the genes of the evolved rules using David Database to identify top significant rules with their involved KEGG pathways or GO-terms. The top rules occupied in a significant number of pathways/GOs are obtained. Frequency of occurrence of the genes in the evolved rules for experimental/treated class is performed to identify potential biomarkers.

## Results and Discussion

In this section, at first, we describe the real datasets that are utilized to verify the performance of our proposed method (i.e., *StatBicRM*). Thereafter, we have performed experiments on the real datasets as well as some artificial datasets. The artificial datasets are made by taking random boolean values. Hence, some related discussions are also included at the end of this section.

### Real Datasets

We have used three real datasets. The datasets are described in Table 1.

**Table 1:** Information of used Real Datasets (DS).

**DS id**	**Dataset information**
DS1	Expression dataset (NCBI ref. id:- GSE10245) of lung cancer subtypes [31], having 40 adenocarcinoma (AC) samples, and 18 squamous cell carcinoma (SCC) samples.
DS2	Expression dataset (NCBI ref. id:- GSE31699) of Uterine Leiomyoma [37], belonging 16 Uterine Leiomyoma tumor (UL) samples and 16 normal myometrial (MM) samples.
DS3	Methylation dataset (NCBI ref. id:- GSE31699) of Uterine Leiomyoma having the 18 UL samples and 18 MM samples.

### Experimental Results and Discussion

**Fig 5:**
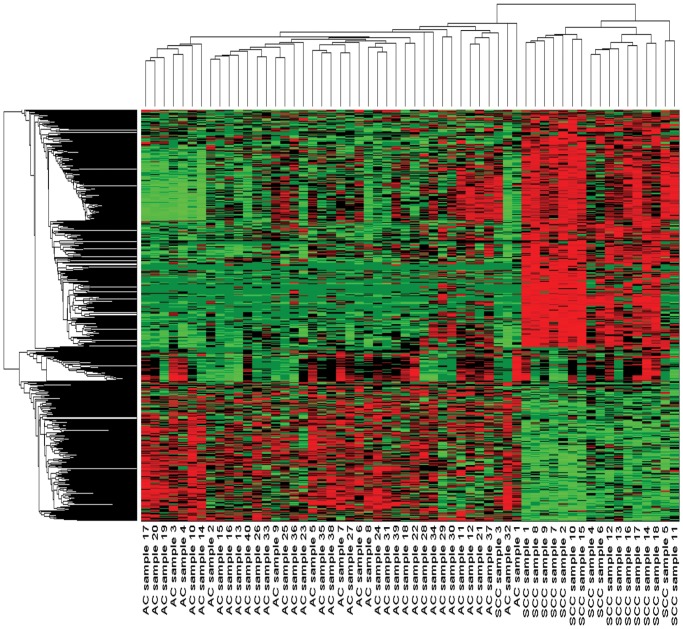
The clustergram of the common differentially expressed genes (by different statistical tests) for DS1. Here, red colour denotes up-regulation of genes across the specific samples/conditions, and green colour denotes down-regulation of genes across the specific samples/conditions.

**Fig 6:**
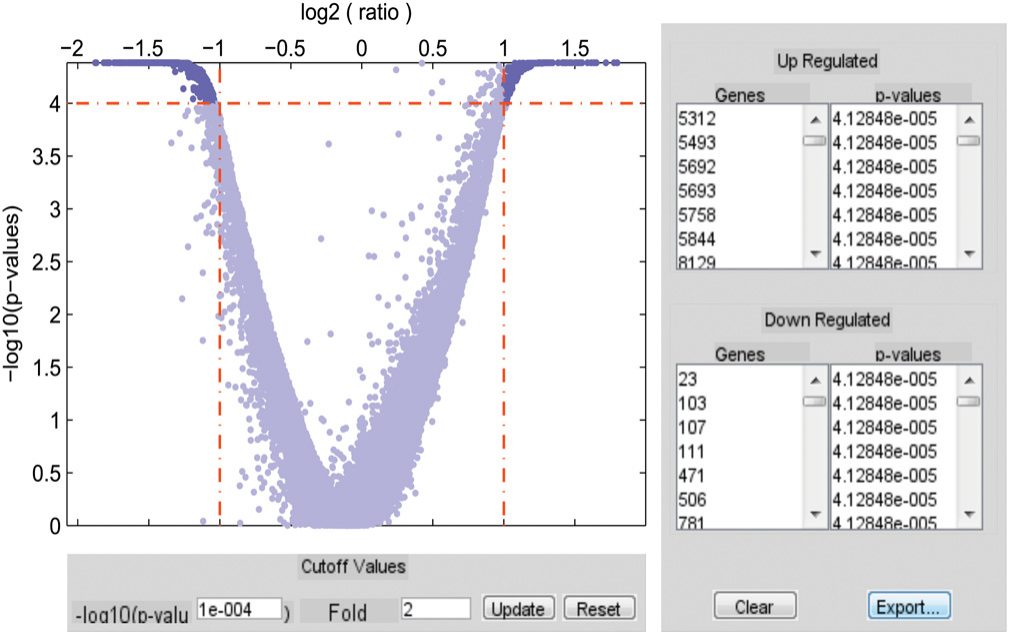
Volcanoplot for identifying differential up and down-regulated genes from Dataset 1 by SAM.

As stated in section Materials and Methods, we have initially applied the statistical filtering strategy (viz., removal of genes having low variance, normalization, normality test, different parametric/non-parametric tests, consecutively) on the (three) real datasets. For the combined dataset (GSE31699), we have firstly identified 13072 common genes that have both expression data (in Dataset 2) as well as methylation data (in Dataset 3). Thereafter, we have found that 18228, 10236 and 8176 genes are following normal distribution, and 24222, 2836 and 4896 genes don’t follow normal distribution for the three datasets, respectively. For DS1, we have identified a total of 1160 differentially expressed genes (viz., ∣*TOTALDESET*_(*N*+*NN*)_∣ = 1160) including ∣*UPDESET*_*N*_∣ = 344, ∣*UPDESET*_*NN*_∣ = 93, ∣*DOWNDESET*_*N*_∣ = 403 and ∣*DOWNDESET*_*NN*_∣ = 320 at 0.0001 p-value cutoff. Table 2 shows this. Similarly, for DS2, we have determined a total of 292 differentially expressed genes (viz., ∣*TOTALDESET*_(*N*+*NN*)_∣ = 292) including ∣*UPDESET*_*N*_∣ = 54, ∣*UPDESET*_*NN*_∣ = 86, ∣*DOWNDESET*_*N*_∣ = 82 and ∣*DOWNDESET*_*NN*_∣ = 70 at 0.05 p-value cutoff. Table 3 represents this. For DS3, we have identified a total of 536 differentially methylated genes (viz., ∣*TOTALDMSET*_(*N*+*NN*)_∣ = 536) including ∣*HYPERDMSET*_*N*_∣ = 74, ∣*HYPERDMSET*_*NN*_∣ = 181, ∣*HYPODMSET*_*N*_∣ = 118 and ∣*HYPODMSET*_*NN*_∣ = 163 at 0.05 p-value cutoff. Table 4 represents this. These genes (i.e., ∣*TOTALDESET*_(*N*+*NN*)_∣ or ∣*TOTALDMSET*_(*N*+*NN*)_∣) are used in the next. Hence, clustergram of the differentially expressed genes for Dataset 1 is shown in Fig. 5. A volcanoplot which is used for identifying up-regulated genes as well as down-regulated genes using SAM for Dataset 1, is presented in Fig. 6.

**Table 2:** Number of differentially expressed genes by different statistical tests for Dataset 1, where #*G*_*up*_, #*G*_*dw*_ denote up and down-regulated genes, respectively. Here, Pearson’s correlation test can not be used as the number of experimental samples is not equal to the number of control samples.

	**parametric tests at 0.0001 p-value cutoff**				**non-parametric tests at 0.0001 p-value cutoff**				
	**t-test**	**Welch’s t-test**	**Bayes’ t-test**	**common**	**Limma**	**SAM**	**Wilcoxon**	**permute**	**common**
#*G*_*up*_	616	586	376	344	115	136	188	176	93
#*G*_*dw*_	619	642	481	403	325	387	615	582	320

**Table 3:** Number of differentially expressed genes by different statistical tests for Dataset 2, where #*G*_*up*_, #*G*_*dw*_ denote up and down-regulated genes, respectively.

	**parametric tests at 0.05 p-value cutoff**					**non-parametric tests at 0.05 p-value cutoff**				
	**t-test**	**Welch’s t-test**	**Bayes t-test**	**Pearson’s correlation**	**common**	**Limma**	**SAM**	**Wilcoxon**	**permute**	**common**
#*G*_*up*_	391	391	329	62	54	86	86	86	86	86
#*G*_*dw*_	576	576	491	97	82	70	70	70	70	70

**Table 4:** Number of differentially methylated genes by different statistical tests for Dataset 3, where #*G*_*hyper*_ and #*G*_*hypo*_ refer to hyper and hypo-methylated genes, respectively.

	**parametric tests at 0.05 p-value cutoff**					**non-parametric tests at 0.05 p-value cutoff**				
	**t-test**	**Welch’s t-test**	**Bayes t-test**	**Pearson’s correlation**	**common**	**Limma**	**SAM**	**Wilcoxon**	**permute**	**common**
#*G*_*hyper*_	652	652	507	87	74	185	185	181	186	181
#*G*_*hypo*_	676	676	600	129	118	165	165	163	166	163

Furthermore, we have prepared an artificial large microarray dataset (denoted by “Dataset 4” or “DS4”) having large number of experimental samples as well as large number of control samples (viz., 100 experimental samples and 100 control samples) and 30000 genes. We have included this dataset into our experiment for validating the performance of classifiers for large number of samples. We have applied our proposed method on the DS4 like DS1, DS2 and DS3. For the DS4, we have obtained that 5348 genes are following normal distribution, and remaining 24462 genes do not follow normal distribution. Thereafter, we have determined a total of 942 differentially expressed genes (viz., ∣*TOTALDESET*_(*N*+*NN*)_∣ = 942) including ∣*UPDESET*_*N*_∣ = 51, ∣*UPDESET*_*NN*_∣ = 532, ∣*DOWNDESET*_*N*_∣ = 23 and ∣*DOWNDESET*_*NN*_∣ = 336 at 0.05 p-value cutoff. Table 5 shows this.

**Table 5:** Number of differentially expressed genes by different statistical tests for the artificial Dataset 4, where #*G*_*up*_, #*G*_*down*_ denote up-regulated and down-regulated genes, respectively.

	**parametric tests at 0.05 p-value cutoff**					**non-parametric tests at 0.05 p-value cutoff**				
	**t-test**	**Welch’s t-test**	**Bayes’ t-test**	**Pearson’s correlation**	**common**	**Limma**	**SAM**	**Wilcoxon**	**permute**	**common**
#*G*_*up*_	90	89	87	56	51	570	573	594	575	532
#*G*_*dw*_	29	29	28	23	23	342	342	365	373	336

**Fig 7:**
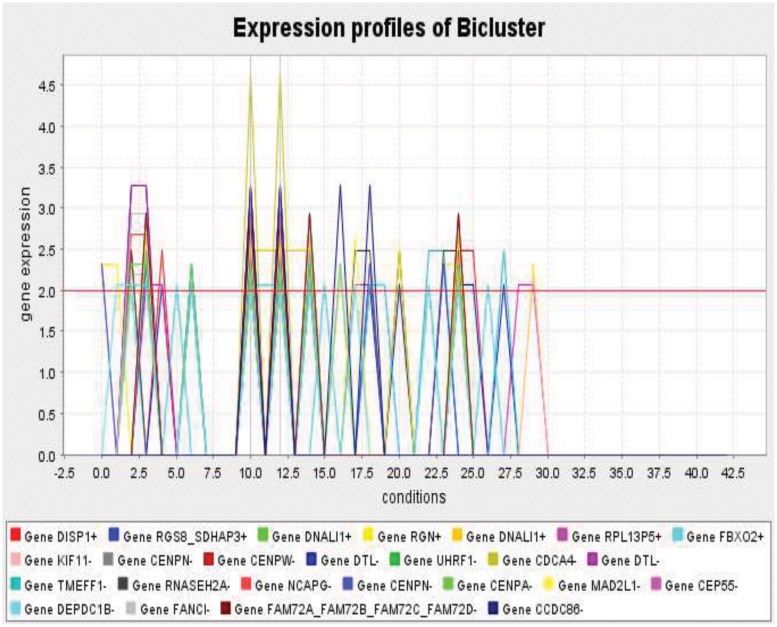
A graphical representation of the gene expression of a maximal homogeneous bicluster (i.e., a *MFCHOI*) over different samples.

**Fig 8:**
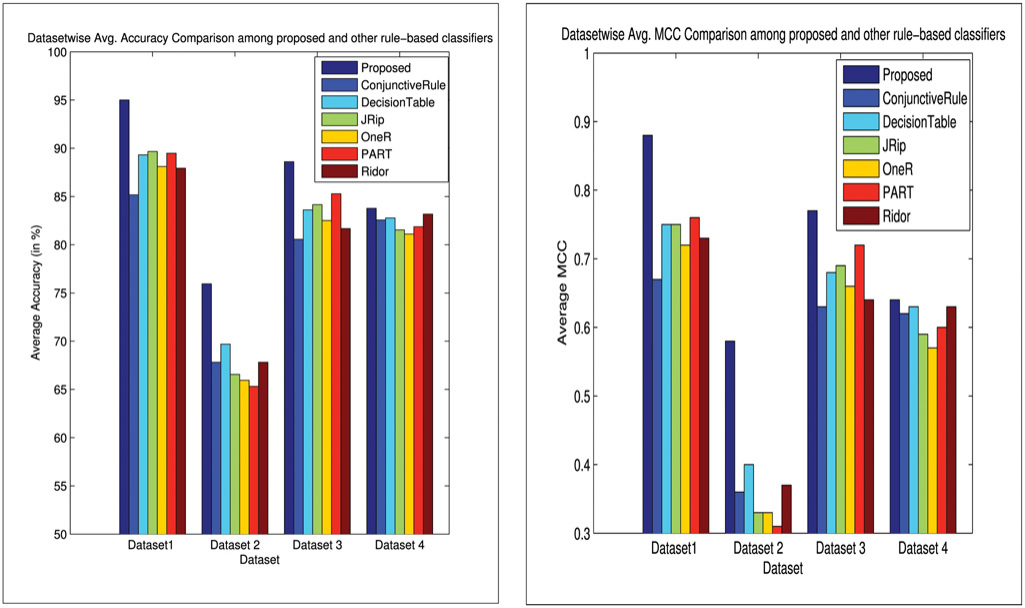
Barcharts: (a) comparison of dataset-wise average accuracies, and (b) comparison of dataset-wise average MCCs, among our proposed and other existing rule-based classifiers for the four datasets.

**Fig 9:**
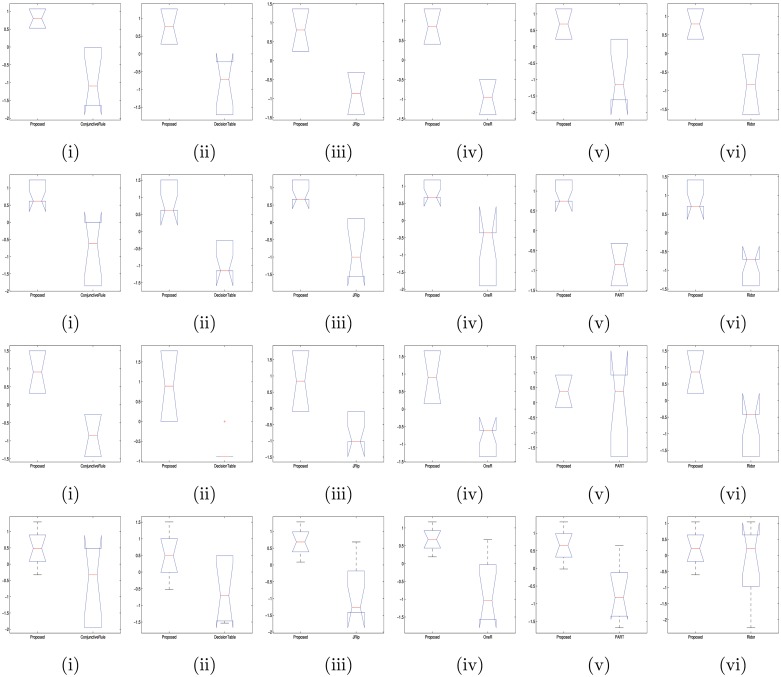
Boxplots of significance tests (i.e., one-way Anova) for identifying level of significances (i.e., p-values) of accuracies between the proposed and other rule-based classifiers (pairwise) for Dataset 1 [in (a).(i-vi)], Dataset 2 [in (b).(i-vi)], Dataset 3 [in (c).(i-vi)] and Dataset 4 [in (d).(i-vi)]; where (i) proposed vs ConjunctiveRule, (ii) proposed vs DecisionTable, (iii) proposed vs JRip, (iv) proposed vs OneR, (v) proposed vs PART and (vi) proposed vs Ridor; (here vertical axis denotes the accuracy of the classifier).

As stated in section Materials and Methods, after statistical testing analysis, we have applied discretization, post-discretization, dividing the data into training and test sets using 4-fold CVs, finding *MFCHOIs* (i.e., maximal biclusters), and extracting the special rules, consecutively. E.g., a bicluster of a few *DE*_*up*_ genes (viz., DISP1, DNALI1, RGN, RPL13P5, FBXO2 etc.) and *DE*_*down*_ genes (viz., KIF11, CENPN, CENPW, DTL, UHRF1, CDCA4 etc.) across a set of two homogeneous conditions (i.e., AC-sample11/AC11 and AC-sample13/AC13) for DS1 is presented in Fig. 7 graphically. After extracting rules, ranking of them, weight assigning, majority voting and two-class classification are performed, consecutively (see section Materials and Methods). In our experiment, we have run 4-fold CVs for 10 times for each dataset. Thereafter, we have calculated average sensitivity, average specificity, average accuracy and average MCC, and finally compared these with the existing rule-based classifiers (presented in Table 6 for DS1, Table 7 for DS2, Table 8 for DS3, and Table 9 for DS4). For each of the four datasets, our proposed method provides the better average accuracy and average MCC than the other six classifiers. It provides 95%, 75.94%, 88.61% and 83.77% accuracies, and 0.88, 0.58, 0.77 and 0.64 MCCs for the four datasets, respectively. For DS1, it provides the best average sensitivity (viz., 99.25%) and average specificity (viz., 85.55%). For DS2, it shows the best average sensitivity (i.e., 98.13%), but its average specificity (i.e., 53.75%) is less than the other classifiers. Similarly, for DS3, it produces the best average sensitivity (i.e., 90.56%), but its average specificity (i.e., 86.67%) is less than some of the four classifiers (viz., PART, JRip, ConjunctiveRule and OneR). For DS4, it produces the best average sensitivity (i.e., 84.13%), but its average specificity (i.e., 83.62%) is less than the “PART” classifier. Fig. 8 shows the comparison of the dataset-wise average accuracies and average MCCs, respectively among our proposed method and the other rule-based classifiers for the four datasets. Thereafter, a statistical significance test (viz., One-way Anova) is performed in between our method and each of the other classifiers pairwise. We have obtained significant p-values for all the pairwise comparisons (i.e., statistical tests) for DS1 and DS2, whereas we have identified that five among the six comparisons are statistically significant for DS3 as well as DS4 (see Fig. 9). The significance level (i.e., p-value) of each comparison is presented in Table 10.

**Table 6:** Comparative performance analysis of the rule-based classifiers on Dataset 1, respectively (at 4-fold CVs repeating for 10 times); where bold font denotes the highest value for each column.

**Rule-based classifier**	**Average sensitivity[%] (s.d.)**	**Average specificity[%] (s.d.)**	**Average accuracy[%] (s.d.)**	**Average MCC (s.d.)**
**Proposed**	**99.25** (1.21)	**85.55** (2.87)	**95** (1.51)	**0.88** (0.035)
**ConjunctiveRule**	88.25 (6.46)	78.33 (15.81)	85.18 (4.00)	0.67 (0.086)
**DecisionTable**	94.75 (2.19)	77.22 (12.13)	89.31 (2.27)	0.75 (0.057)
**JRip**	94.25 (1.21)	79.45 (6.95)	89.66(1.41)	0.75 (0.037)
**OneR**	92.5 (2.04)	78.33 (8.05)	88.11 (1.51)	0.72 (0.039)
**PART**	92 (2.58)	83.89 (4.86)	89.48 (3.19)	0.76 (0.074)
**Ridor**	91.75 (5.90)	79.44 (17.18)	87.93 (2.82)	0.73 (0.072)

**Table 7:** Comparative performance analysis of the rule-based classifiers on Dataset 2, respectively (at 4-fold CVs repeating for 10 times); where bold font denotes the highest value for each column.

**Rule-based classifier**	**Average sensitivity[%] (s.d.)**	**Average specificity[%] (s.d.)**	**Average accuracy[%] (s.d.)**	**Average MCC (s.d.)**
**Proposed**	**98.13** (3.02)	53.75 (3.23)	**75.94** (1.51)	**0.58** (0.037)
**ConjunctiveRule**	71.88 (8.46)	63.75 (8.23)	67.81 (3.91)	0.36 (0.081)
**DecisionTable**	76.88 (3.02)	62.5 (5.10)	69.69 (1.51)	0.40 (0.027)
**JRip**	70.63 (3.02)	62.5 (5.10)	66.56 (3.92)	0.33 (0.080)
**OneR**	73.13 (3.02)	58.75 (10.70)	65.93 (4.53)	0.33 (0.087)
**PART**	66.25 (6.04)	**64.38** (7.83)	65.31 (2.74)	0.31 (0.057)
**Ridor**	76.88 (3.02)	58.75 (3.23)	67.81 (1.51)	0.37 (0.031)

**Table 8:** Comparative performance analysis of the rule-based classifiers on Dataset 3, respectively (at 4-fold CVs repeating for 10 times); where bold font denotes the highest value for each column.

**Rule-based classifier**	**Average sensitivity[%] (s.d.)**	**Average specificity[%] (s.d.)**	**Average accuracy[%] (s.d.)**	**Average MCC (s.d.)**
**Proposed**	**90.56** (2.68)	86.67 (2.87)	**88.61** (2.43)	**0.77** (0.048)
**ConjunctiveRule**	70.56 (2.68)	90.56 (6.95)	80.56 (2.27)	0.63 (0.062)
**DecisionTable**	84.44 (3.51)	82.78 (4.86)	83.61 (0.88)	0.68 (0.019)
**JRip**	75.56 (2.87)	92.78 (2.68)	84.16 (1.34)	0.69 (0.027)
**OneR**	76.67 (7.31)	88.33 (4.86)	82.50 (1.33)	0.66 (0.014)
**PART**	76.11 (13.11)	**94.44** (0.00)*	85.28 (6.55)	0.72 (0.113)
**Ridor**	83.33 (4.54)	80.00 (9.51)	81.67 (2.68)	0.64 (0.048)

* This standard deviation of specificity is coming to be zero. On investigation, we have identified a particular datapoint belonging to normal class in Dataset 3 for which the “PART” classifier as well as the other classifiers including the proposed one are producing always false positive result.

**Table 9:** Comparative performance analysis of the rule-based classifiers on Dataset 4, respectively (at 4-fold CVs repeating for 10 times); where bold font denotes the highest value for each column.

**Rule-based classifier**	**Average sensitivity[%] (s.d.)**	**Average specificity[%] (s.d.)**	**Average accuracy[%] (s.d.)**	**Average MCC (s.d.)**
**Proposed**	**84.13** (2.37)	83.62 (0.37)	**83.77** (0.67)	**0.64** (0.02)
**ConjunctiveRule**	83.47 (2.32)	82.12 (0.98)	82.56 (1.22)	0.62 (0.04)
**DecisionTable**	84.06 (3.82)	81.32 (0.78)	82.78 (0.87)	0.63 (0.02)
**JRip**	79.12 (2.89)	83.37 (0.97)	81.55 (1.37)	0.59 (0.04)
**OneR**	79.63 (3.48)	81.75 (1.48)	81.10 (1.78)	0.57 (0.06)
**PART**	80.95 (2.96)	**83.97** (0.93)	81.86 (1.27)	0.60 (0.04)
**Ridor**	84.07 (2.57)	82.56 (1.05)	83.17 (1.42)	0.63 (0.05)

**Table 10:** p-value of Anova 1 between the avg. accuracies of the proposed and other classifiers (pairwise) in DS1, DS2, DS3 and DS4 (where ‘S’ and ‘NS’ refer to significant (p-value ≤ 0.05) and non-significant (p-value > 0.05) p-values respectively).

**Group**	**p-value in DS1**	**p-value in DS2**	**p-value in DS3**	**p-value in DS4**
**Proposed vs ConjunctiveRule**	2.41e-06 (S)	8.68e-06 (S)	4.53e-07 (S)	0.0139(S)
**Proposed vs DecisionTable**	3.40e-06 (S)	2.88e-08 (S)	8.93e-06 (S)	0.0106(S)
**Proposed vs JRip**	1.78e-07 (S)	1.36e-06 (S)	8.16e-05 (S)	0.0002(S)
**Proposed vs OneR**	6.53e-09 (S)	3.22e-06 (S)	1.68e-06 (S)	0.0003(S)
**Proposed vs PART**	0.0001 (S)	2.89e-09 (S)	0.1491 (NS)	0.0005(S)
**Proposed vs Ridor**	1.57e-06 (S)	4.81e-10 (S)	9.83e-06 (S)	0.2497(NS)

**Fig 10:**
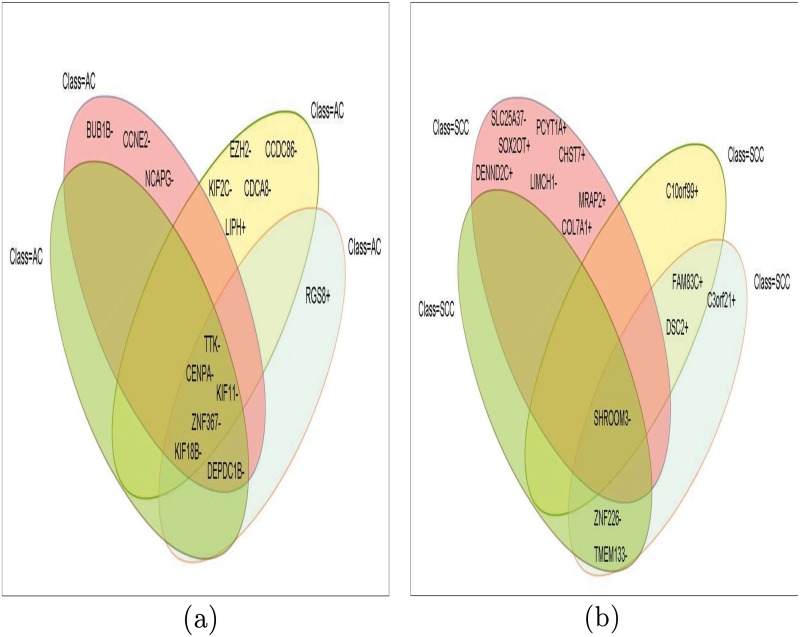
Two examples of how significant biomarkers are identified from the maximal homogeneous biclusters (i.e., *MFCHOI*) for each class-label for each dataset. Here, we are shown intersection of only four maximal homogeneous biclusters for (a) the class-label *AC* and (b) the class-label *SCC*, individually (for Dataset 1). For the class *AC*, CENPA-, TTK-, KIF11-, KIF18B- and ZNF367- are the top frequent genes as they exist in the four biclusters (see (a)); similarly, for the class *SCC*, SHROOM3- is top frequent gene as it exists in the four biclusters (see (b)).

For DS1, we have obtained a set of rules, where some of these are of cancer subtype AC, and remaining of these are of cancer subtype SCC (i.e., these rules have ‘class = AC’/‘class = SCC’ in their consequent parts). We have produced a list of top frequent genes occurring in the evolved rules of each cancer subtype. We have identified **‘CENPA-’** as top frequent gene for cancer subtype *AC*. But, **‘CENPA-’** is not found in any evolved rule of cancer subtype *SCC*. Therefore, it is important for cancer subtype *AC*. Some literature evidences have been found in [29, 30] related to it. (According to the dataset, fold change of CENPA gene is 0.791 which indicates down-regulation of it as this value is less than 1; and p-values of it are 5.00E-05 in SAM, 2.53E-05 in Limma, 7.46E-07 in permuted t-test and 8.42E-06 in Wilcoxon ranksum test which indicate that it is a differentially expressed gene.) Besides that, we have also found literature-match of TTK in [31]. CENPN, KIF2C, EZH2 genes are also found in literatures [32, 33] related to *AC*. Similarly, ‘SHROOM3-’ is the top frequent gene for *SCC*. In DS2, we have identified a set of rules, where some of them are of *UL* class (i.e., tumor class), and remaining rules of them are of *MM* class (i.e., normal class). We have found some literature documentations about PRL in [34], TRPC6 in [35], and IGF2 in [36]. Similarly, for DS3, we have identified some set of rules, where some of them are of *UL* class (i.e., tumor class), and remaining rules of them are of *MM* class (i.e., normal class). The top 10 frequent genes for the two classes for the datasets are shown in Table 11, respectively. Some evidence of PLP1 in forming the tumor in [37] and similar documentary support of TRPM2 in forming the tumor are found in [38]. In Fig. 10 depicts two examples of how significant biomarkers are identified for each class-label for each real dataset.

**Table 11:** Top 10 frequent genes in evolved rules of the two class-labels for DS1, DS2 and DS3, respectively. *Rule*_*experimental*_ and *Rule*_*control*_ denote the set of the evolved rules of experimental class-label, and the set of the evolved rules of control class-label, respectively.

	**DS1**	**DS2**	**DS3**
For *Rule*_*experimental*_	**CENPA-**	**MCM4+**	**GZMH-**
	**TTK-**	**PRL+**	**TRPM2-**
	**CENPN-**	**FBXO33-**	**LHCGR+**
	**KIF2C-**	**NUAK1-**	**IQCF2-**
	**EZH2-**	**JAG1-**	**BSG+**
	**CA12-**	**EGFL6+**	**SCN4B+**
	**RGN+**	**CDC34+**	**HCG9+**
	**NCAPG-**	**TRPC6+**	**C1orf158-**
	**RNASEH2A-**	**IGF2+**	**PLP1-**
	**RPL13P5+**	**PSCD1-**	**SMPD2-**
For *Rule*_*control*_	**SHROOM3-**	**MEIS3-**	**PRSS8-**
	**CMTM8-**	**AOX1+**	**NAV1+**
	**ZNF226-**	**ZNF217+**	**LYZL2-**
	**UGT1A8+/UGT1A9+**	**GFOD1+**	**FYB+**
	**MRAP2+**	**PRRG1+**	**EML4-**
	**SOX2OT+**	**MTMR4-**	**LHPP+**
	**CXXC5-**	**SERPINB1+**	**CCDC13-**
	**XKR8-**	**FHL5+**	**TEK-**
	**C10orf99+**	**ACSL5+**	**INHBE-**
	**FAM83C+**	**LIFR+**	**S100A16+**

Besides that, KEGG pathway and GO analysis have been performed on the genes of the evolved rules for the three real datasets using David Database. The significant KEGG pathways, GO:BP, GO:CC and GO:MF terms are presented in Table 12. Some top important rules w.r.t. their existing KEGG pathways, GO:BPs, GO:CCs and GO:MFs, individually are shown in Table 13, Table 14 and Table 15 for the three real datasets (i.e., DS1, DS2 and DS3), respectively. Here, an important rule w.r.t. their existing pathways/GO-terms refers to such a rule whose all the genes (i.e., all genes of consequent part) involve together in maximum number of pathways/GO-terms. The details about the significant KEGG pathways, GO:BPs, GO:CCs and GO:MFs are presented in S1 File, S2 File and S3 File for the three datasets, respectively. S2 Text shows the top 15 rules from the three real datasets.

**Table 12:** KEGG pathway, GO:BP, GO:CC and GO:MF analysis of corresponding genes of the evolved rules from the three datasets. *Here, ‘satisfiable rule’ or SRule by some KEGG-pathway(i.e., Path)/GO:BP/GO:CC/GO:MF means that all the genes (i.e., antecedent) of the rule are occurred together in the pathway/Go-term*.

**DS**		**Pathway/GO-BP/GO-CC/GO-MF**	**p-value**	**#Gene**	**Genes**	**#SRule**	**SRule ids**
**DS1**	**Path**	**hsa04120:Ubiquitin mediated proteolysis**	0.0386	7	MGRN1, FBXO2, KLHL13, DDB2, RHOBTB2, MID1, UBE2S	1	rule id 5233
	**GO:BP**	**GO:0022402 cell cycle process**	8.69E-10	34	PRC1, BLM, TTK, PKMYT1, CEP55, AURKB, RHOU, GTSE1, SPC24, KIF2C, CDCA8, NCAPH, NCAPG, CENPA, SKA1, ZWILCH, TXNL4B, CDK1 etc.	21	rule id 327, 2231, 2232, 2914, 7360 etc.
		**GO:0000278 mitotic cell cycle**	1.02E-11	30	PRC1, BLM, TTK, PKMYT1, SPC24, KIF15, BIRC5, CENPE, NDC80, SMC2, CDK2, MAD2L1, TIMELESS, PLK1, BUB1B, SETD8 etc.	19	rule id 327, 2231, 2232, 2914, 7360 etc.
		**GO:0022403 cell cycle phase**	6.04E-12	32	PRC1, BLM, TTK, PKMYT1, CEP55, AURKB, RHOU, GTSE1, BIRC5, CENPE, NDC80, SMC2, CDK2, MAD2L1, TIMELESS, PLK1, BUB1B, RAD54B etc.	18	rule id 327, 2231, 2232, 2914, 7360 etc.
		**GO:0007017 microtubule-based process**	4.86E-04	14	KIF11, PRC1, KIF15, KIF18B, TTK, NDC80, CENPE, MID1, MARK1, GTSE1, KIF2C, CENPA, BUB1B, KIF13B	17	rule id 327, 2232, 7360 etc.
	**GO:CC**	**GO:0043228 non-membrane-bounded organelle**	5.43E-05	70	MTSS1, FOSL2, PRC1, CEP78, TTK, AURKB, SENP5, RHOU, GTSE1, SLC1A4, KIF2C, CDCA8, FRMD6, PBXIP1, FANCI, SNTB1, KIF13B, CDK1, MYO6, KIF11 etc.	85	rule id 151, 253, 298, 327, 415, 888, 1261, 1462, 1970, 2232 etc.
		**GO:0043232 intracellular non-membrane-bounded organelle**	5.43E-05	70	MTSS1, FOSL2, PRC1, CEP78, TTK, AURKB, SENP5, RHOU, GTSE1, SLC1A4, KIF2C, CDCA8, FRMD6 etc.	85	rule id 151, 253, 298, 327, 415, 888, 1261, 1462, 1970, 2232 etc.
		**GO:0044459 plasma membrane part**	0.0128	52	DLC1, IL27RA, TSPAN4, RHOU, SLC1A4, FRMD6, CD44, LTB4R, SNTB1, CEACAM6, SLC22A3, RAB27A, ARHGEF4, ICAM1, PLD1, MYO6, LIFR etc.	36	rule id 212, 625, 1876, 6051 etc.
	**GO:MF**	**GO:0000166 nucleotide binding**	0.0015	56	ACOX2, CTPS, PKMYT1, TTK, AURKB, RHOU, KIF2C, MCM8, LTB4R, ACAD8, RAB27B, ACAD9, RAB27A, KIF13B, NMNAT3, CDK1, MYO6, KIF11, LIMK2, KIF15, MCM4, MBD1, MCM5, CDK2, etc.	51	rule id 254, 327, 339, 344, 494, 639, 643 etc.
		**GO:0001883 purine nucleoside binding**	4.95E-04	45	ACOX2, FGFR2, BLM, CTPS, TTK, PKMYT1, AURKB, ADA, KIF2C, IGF1R, MCM8, STK32A, ACAD8, ACAD9, KIF13B, MYO5C, NMNAT3, CDK1, MYO6, KIF11, MKI67, LIMK2, KIF15, ATP11B etc.	39	rule id 254, 327, 339, 344, 494, 639, 643 etc.
		**GO:0001882 nucleoside binding**	5.72E-04	45	ACOX2, FGFR2, BLM, CTPS, TTK, PKMYT1, AURKB, ADA, KIF2C, IGF1R, MCM8, STK32A, ACAD8, ACAD9, KIF13B, MYO5C, NMNAT3, CDK1, MYO6, KIF11, MKI67, LIMK2, IPPK, UBE2S, ABCC5 etc.	39	rule id 254, 327, 339, 344, 494, 639, 643 etc.
**DS2**	**Path**	**hsa00982:Drug metabolism**	9.79E-04	5	GSTA4, FMO2, AOX1, GSTO2, MGST1	1	rule id 12
	**BP**	**GO:0042127 regulation of cell proliferation**	0.0341	9	CEBPA, TNFSF4, BAX, SERPINE1, LIFR, IGF2, JAG1, CD24, PRL	4	rule id 78, 95, 145, 390
		**GO:0032583 regulation of gene-specific transcription**	0.0039	5	CEBPA, TNFSF4, SMARCB1, PSRC1, IGF2	3	rule id 52, 78, 225
	**CC**	**GO:0005576 extracellular region**	0.0015	20	TNFSF4, EGFL6, MMP9, APOC1, LIFR, GGH, IGF2, JAG1, MMP2, CHRDL1, PRRG1, PTGDS, C1QTNF4, SERPINE1, PECAM1, SERPINA3, C1QL1, GDF15, GFOD1, PRL	6	rule id 50,81,82,87,95,246
**DS3**	**Path**	**hsa04060:Cytokine-cytokine receptor interaction**	1.69E-04	12	EGFR, IFNA21, CCR1, TNFSF12, IFNA1, IL23A, IL20RA, CCL3L1, INHBE, TNFRSF18, TNFSF12-TNFSF13, IFNGR2, IFNA17	1	rule id 177
	**GO:BP**	**GO:0006952 defense response**	1.88E-09	29	IFNA21, S100A8, CCR1, BNIP3, HTN3, CD74, CFHR1, APOA4, REG3A, IFNA1, IL23A, SAA2, CCL3L1, SAA1, REG3G, CFHR5, IL1RL1, DEFB103A, SCUBE1, RNASE6 etc.	12	rule id 7, 144, 613, 617, 653, 654, 784, 822, 1067, 1182, 2293, 2342
		**GO:0006955 immune response**	8.71E-04	20	FYB, IL1RL1, SLA2, CCR1, IGJ, CD300E, BNIP3, TNFSF12, C4BPA, CD74, CLEC4M, APOA4, CFHR1, CYBA, IL23A, CCL3L1, LYST, DEFA1, TNFSF12-TNFSF13, TREM1, CFHR5	12	rule id 7, 349, 350, 351, 387, 654, 784, 1182, 1674, 2293, 2342, 2361
		**GO:0003012 muscle system process**	0.0048	8	CYBA, CALD1, MYH3, SLMAP, MYH4, ACTN2, SCN5A, CASQ2	4	rule id 47, 138, 333, 2296
		**GO:0006936 muscle contraction**	0.0118	7	CALD1, MYH3, SLMAP, MYH4, ACTN2, SCN5A, CASQ2	4	rule id 47, 138, 333, 2296
	**GO:CC**	**GO:0005886 plasma membrane**	0.0090	67	TEX101, STEAP4, NEURL, LHCGR, F2RL1, FCRL2, TNFSF12, KCNIP4, CALB2, FCRL3, APOB, SLMAP, ERAS, CALCRL, IFNGR2, EGFR, BSG, SLA2, SCUBE1, ACTN2, CACNG3, OR1D2, FLNA, TRPM2 etc.	91	rule id 126, 144, 155, 272, 321, 338, 339, 351, 385, 416 etc.
		**GO:0044459 plasma membrane part**	0.0112	43	PKHD1, CCR1, LHCGR, F2RL1, TRHR, PANX3, CLDN11, TNFSF12, CD74, CALB2, SORBS3, SLMAP, TEK, ERAS, CALCRL, IFNGR2, SCN5A, EGFR, TRPM2, KCNK3, CLEC4M etc.	43	rule id 27, 28, 126, 144, 301, 339, 351, 385, 513, 514 etc.
		**GO:0005576 extracellular region**	4.55E-09	59	IFNA21, LHCGR, MMP27, TNFSF12, HTN3, APOA4, CFHR1, CFHR2, REG3A, APOB, OLFML3, SAA2, SERPINE2, SAA1, CCL3L1, CREG1, ANGPT1, REG3G, CFHR5, EGFR, NODAL, DEFA1 etc.	40	rule id 151, 180, 191, 346, 349, 350, 351, 486, 515, 517 etc.
	**GO:MF**	**GO:0046983 protein dimerization activity**	0.0029	16	EGFR, S100A16, SCUBE1, TRHR, LHCGR, NFS1, BNIP3, DSCAML1, ACTN2, FLNA, APOA4, CYBA, APOB, BOK, TFAP2E, CRYBB2	3	rules 1040, 1176, 2358
		**GO:0019955 cytokine binding**	0.0112	6	IL1RL1, IL20RA, CCR1, TNFRSF18, IFNGR2, CD74	2	rule id 177, 2342

**Table 13:** Some top important rules w.r.t. their existing KEGG pathways/GO:BPs/GO:CCs/GO:CCs/GO:MFs in Dataset 1.

**Rule**	**#Pathway**	**Pathways**
{FBXO2+, DDB2-⇒ class = AC}	1	hsa04120:Ubiquitin mediated proteolysis
**Rule**	**#GO:BP**	**GO:BPs**
{KIF11-, BUB1B- ⇒ class = AC }	14	GO:0000279 M phase, GO:0000280 nuclear division, GO:0007067 mitosis, GO:0022403 cell cycle phase, GO:0000087 M phase of mitotic cell cycle, GO:0007049 cell cycle, GO:0000278 mitotic cell cycle, GO:0048285 organelle fission, GO:0051301 cell division, GO:0022402 cell cycle process, GO:0007010 cytoskeleton organization, GO:0007017 microtubule-based process, GO:0000226 microtubule cytoskeleton organization, GO:0007051 spindle organization
{KIF11-, TTK- ⇒ class = AC }	10	GO:0000279 M phase, GO:0022403 cell cycle phase, GO:0007049 cell cycle, GO:0000278 mitotic cell cycle, GO:0022402 cell cycle process, GO:0007010 cytoskeleton organization, GO:0007017 microtubule-based process, GO:0007052 mitotic spindle organization, GO:0000226 microtubule cytoskeleton organization, GO:0007051 spindle organization
{KIF11-, TIMELESS- ⇒ class = AC }	10	GO:0000279 M phase, GO:0000280 nuclear division, GO:0007067 mitosis, GO:0022403 cell cycle phase, GO:0000087 M phase of mitotic cell cycle, GO:0007049 cell cycle, GO:0000278 mitotic cell cycle, GO:0048285 organelle fission, GO:0051301 cell division, GO:0022402 cell cycle process
{NCAPH+, AURKB+, KIF15+ ⇒ class = SCC }	9	GO:0000279 M phase, GO:0000280 nuclear division, GO:0007067 mitosis, GO:0022403 cell cycle phase, GO:0000087 M phase of mitotic cell cycle, GO:0007049 cell cycle, GO:0000278 mitotic cell cycle, GO:0048285 organelle fission, GO:0022402 cell cycle process
**Rule**	**#GO:CC**	**GO:CCs**
{CENPN-, ZWILCH- ⇒ class = AC}	9	GO:0000793 condensed chromosome, GO:0000779 condensed chromosome and centromeric region, GO:0000775 chromosome and centromeric region, GO:0000777 condensed chromosome kinetochore, GO:0000776 kinetochore, GO:0044427 chromosomal part, GO:0005694 chromosome, GO:0043228 non-membrane-bounded organelle, GO:0043232 intracellular non-membrane-bounded organelle
{CENPN-, CENPA- ⇒ class = AC}	9	GO:0000793 condensed chromosome, GO:0000779 condensed chromosome and centromeric region, GO:0000775 chromosome and centromeric region, GO:0000777 condensed chromosome kinetochore, GO:0000776 kinetochore, GO:0044427 chromosomal part, GO:0005694 chromosome, GO:0043228 non-membrane-bounded organelle, GO:0043232 intracellular non-membrane-bounded organelle
{CENPN-, CENPM- ⇒ class = AC}	9	GO:0000793 condensed chromosome, GO:0000779 condensed chromosome and centromeric region, GO:0000775 chromosome and centromeric region, GO:0000777 condensed chromosome kinetochore, GO:0000776 kinetochore, GO:0044427 chromosomal part, GO:0005694 chromosome, GO:0043228 non-membrane-bounded organelle, GO:0043232 intracellular non-membrane-bounded organelle
**Rule**	**#GO:MF**	**GO:MFs**
{SMC2-, TTK- ⇒ class = AC}	9	GO:0001883 purine nucleoside binding, GO:0001882 nucleoside binding GO:0030554 adenyl nucleotide binding, GO:0000166 nucleotide binding GO:0017076 purine nucleotide binding, GO:0005524 ATP binding GO:0032559 adenyl ribonucleotide binding, GO:0032555 purine ribonucleotide binding, GO:0032553 ribonucleotide binding
{TTK-, KIF2C- ⇒ class = AC}	9	GO:0001883 purine nucleoside binding, GO:0001882 nucleoside binding GO:0030554 adenyl nucleotide binding, GO:0000166 nucleotide binding GO:0017076 purine nucleotide binding, GO:0005524 ATP binding GO:0032559 adenyl ribonucleotide binding, GO:0032555 purine ribonucleotide binding, GO:0032553 ribonucleotide binding
{KIF2C-, IGF1R- ⇒ class = AC}	9	GO:0001883 purine nucleoside binding, GO:0001882 nucleoside binding GO:0030554 adenyl nucleotide binding, GO:0000166 nucleotide binding GO:0017076 purine nucleotide binding, GO:0005524 ATP binding GO:0032559 adenyl ribonucleotide binding, GO:0032555 purine ribonucleotide binding, GO:0032553 ribonucleotide binding
{SMC2-, TTK-, KIF2C- ⇒ class = AC}	9	GO:0001883 purine nucleoside binding, GO:0001882 nucleoside binding GO:0030554 adenyl nucleotide binding, GO:0000166 nucleotide binding GO:0017076 purine nucleotide binding, GO:0005524 ATP binding GO:0032559 adenyl ribonucleotide binding, GO:0032555 purine ribonucleotide binding, GO:0032553 ribonucleotide binding
{TTK-, SMC2-, CTPS- ⇒ class = AC}	9	GO:0001883 purine nucleoside binding, GO:0001882 nucleoside binding GO:0030554 adenyl nucleotide binding, GO:0000166 nucleotide binding GO:0017076 purine nucleotide binding, GO:0005524 ATP binding GO:0032559 adenyl ribonucleotide binding, GO:0032555 purine ribonucleotide binding, GO:0032553 ribonucleotide binding

**Table 14:** Some top important rules w.r.t. their existing KEGG pathways/GO:BPs/GO:CCs in Dataset 2. Here, we have got no such significant rule w.r.t. their existing GO:MFs for the dataset.

**Rule**	**#Pathway**	**Pathways**
{AOX1+, GSTA4- ⇒ class = normal}	1	hsa00982:Drug metabolism
**Rule**	**#GO:BP**	**GO:BPs**
{AOX1+, GSTA4- ⇒ class = normal}	2	GO:0032583 regulation of gene-specific transcription, GO:0042127 regulation of cell proliferation
{IGF2+, PRL+ ⇒ class = tumor}	1	GO:0042127 regulation of cell proliferation
{IGF2+, PRL+ ⇒ class = tumor}	1	GO:0042127 regulation of cell proliferation
{IGF2+, PRL+ ⇒ class = tumor}	1	GO:0032583 regulation of gene-specific transcription
{IGF2+, PRL+ ⇒ class = tumor}	1	GO:0042127 regulation of cell proliferation
{IGF2+, PRL+ ⇒ class = tumor}	1	GO:0032583 regulation of gene-specific transcription
**Rule**	**#GO:CC**	**GO:CCs**
{IGF2+, PTGDS- ⇒ class = tumor}	3	GO:0005576 extracellular region, GO:0031090 organelle membrane, GO:0005783 endoplasmic reticulum
{IGF2+, EGFL6+ ⇒ class = tumor}	2	GO:0005576 extracellular region, GO:0005615 extracellular space
{PRRG1+, SERPINE1+ ⇒ class = normal}	1	GO:0005576 extracellular region
{CHRDL1+, JAG1- ⇒ class = tumor}	1	GO:0005576 extracellular region
{IGF2+, PRL+ ⇒ class = tumor}	1	GO:0005576 extracellular region
{SERPINE1+, GFOD1+ ⇒ class = normal}	1	GO:0005576 extracellular region
{JAG1-, PECAM1- ⇒ class = tumor}	1	GO:0005576 extracellular region

**Table 15:** Top important rules w.r.t. their existing KEGG pathways/GO:BPs/GO:CCs/GO:MFs in Dataset 3.

**Rule**	**#Pathway**	**Pathways**
{IL20RA+, CCR1+ ⇒ class = tumor}	1	hsa04060:Cytokine-cytokine receptor interaction
**Rule**	**#GO:BP**	**GO:BPs**
{CYBA+, C4BPA- ⇒ class = tumor}	5	GO:0006952 defense response, GO:0006954 inflammatory response, GO:0009611 response to wounding, GO:0006955 immune response, GO:0045087 innate immune response
{LYST+, BNIP3+ ⇒ class = tumor}	4	GO:0006952 defense response, GO:0009615 response to virus, GO:0006955 immune response, GO:0002252 immune effector process
{CFHR5-, REG3A- ⇒ class = tumor}	4	GO:0006952 defense response, GO:0006954 inflammatory response, GO:0009611 response to wounding, GO:0002526 acute inflammatory response
{CCR1+, CFHR5- ⇒ class = tumor}	4	GO:0006952 defense response, GO:0006954 inflammatory response, GO:0009611 response to wounding, GO:0006955 immune response
**Rule**	**#GO:CC**	**GO:CCs**
{MST1R+, TNFSF12/TNFSF13+ ⇒ class = tumor}	4	GO:0031226 intrinsic to plasma membrane, GO:0005886 plasma membrane, GO:0005887 integral to plasma membrane, GO:0044459 plasma membrane part
{MST1R+, CCR1+, TNFSF12/TNFSF13+ ⇒ class = tumor}	4	GO:0031226 intrinsic to plasma membrane, GO:0005886 plasma membrane, GO:0005887 integral to plasma membrane, GO:0044459 plasma membrane part
{LHCGR+, SLMAP+ ⇒ class = tumor}	4	GO:0031226 intrinsic to plasma membrane, GO:0005886 plasma membrane, GO:0005887 integral to plasma membrane, GO:0044459 plasma membrane part
{CYBA+, MST1R+ ⇒ class = tumor}	4	GO:0031226 intrinsic to plasma membrane, GO:0005886 plasma membrane, GO:0005887 integral to plasma membrane, GO:0044459 plasma membrane part
{TRPM2-, SMPD2- ⇒ class = tumor}	4	GO:0031226 intrinsic to plasma membrane, GO:0005886 plasma membrane, GO:0005887 integral to plasma membrane, GO:0044459 plasma membrane part
{SCN4B+, TRPM2- ⇒ class = tumor}	4	GO:0031226 intrinsic to plasma membrane, GO:0005886 plasma membrane, GO:0005887 integral to plasma membrane, GO:0044459 plasma membrane part
{MST1R+, CALCRL+ ⇒ class = tumor}	4	GO:0031226 intrinsic to plasma membrane, GO:0005886 plasma membrane, GO:0005887 integral to plasma membrane, GO:0044459 plasma membrane part
{S100A16+, MTNR1A-, NODAL- ⇒ class = normal}	4	GO:0031226 intrinsic to plasma membrane, GO:0005886 plasma membrane, GO:0005887 integral to plasma membrane, GO:0044459 plasma membrane part
{TRPM2-, SMPD2-, UGT1A10-⇒ class = tumor}	4	GO:0031226 intrinsic to plasma membrane, GO:0005886 plasma membrane, GO:0005887 integral to plasma membrane, GO:0044459 plasma membrane part
{SLMAP+, CCR1+ ⇒ class = tumor}	4	GO:0031226 intrinsic to plasma membrane, GO:0005886 plasma membrane, GO:0005887 integral to plasma membrane, GO:0044459 plasma membrane part
**Rule**	**#GO:MF**	**GO:MFs**
{BSG+, CLEC4M- ⇒ class = tumor}	2	GO:0005529 sugar binding, GO:0030246 carbohydrate binding

**Fig 11:**
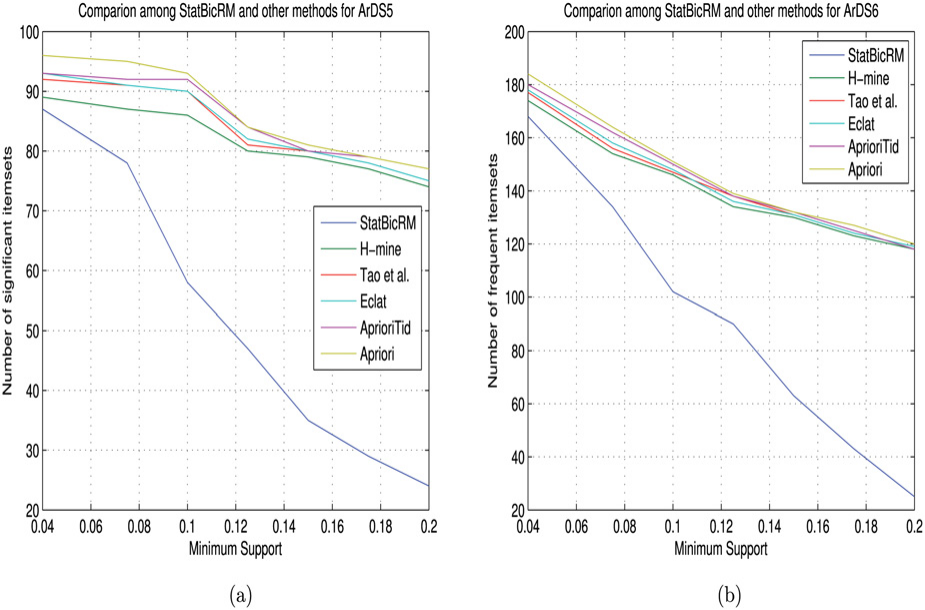
Comparison of number of significant itemsets between *StatBicRM* and other existing ARM methods at different minimum support for the two artificial datasets (viz., *ArDS*5 and *ArDS*6). “Significant itemset” refers to *MFCHOI* for *StatBicRM*, and *FI* for the other methods.

Furthermore, we have compared our proposed rule mining algorithm with the existing ARM algorithms (viz., Traditional Apriori, AprioriTid, Eclat, Tao et al. and H-mine). But, since the number of genes of the datasets are high (i.e., greater than 250), and the other techniques generate frequent itemsets, therefore those methods fails to work on DS1 and DS4, and take long time to execute on DS2 and DS3 (i.e., nearly 5 hours or more) whereas our method can work efficiently on them as our method extracts maximal frequent closed homogeneous itemsets (*MFCHOIs*) using the biclustering technique. Therefore, we have made two artificial binary datasets which are prepared by taking random binary digits (namely 1 or 0). One dataset (denoted by *ArDS*5) among them has 100 genes and 60 samples (i.e., a total of 30 experimental samples and 30 control samples), and other dataset (denoted by *ArDS*6) includes 200 genes and 60 samples (i.e., a total of 30 experimental samples and 30 control samples). Thereafter, we have applied the binary matrix as input of the BiMax biclustering. For each of *ArDS*5 and *ArDS*6, we have observed that our proposed method produces much less number of significant non-redundant itemsets than the other rule mining algorithms as our proposed method determines the maximal frequent closed homogeneous itemsets (*MFCHOIs*) which are proper subsets of frequent itemsets (FIs); i.e., *MFCHOI* ⊂ *FI*. Furthermore, as a single rule is extracted from a single significant itemset (i.e., *MFCHOI*) in our method, thus the number of evolved rules are same with the number of significant itemsets (i.e., *MFCHOIs*). Therefore, the evolved rules in our method are much less in number than the other methods. Thus, elapsed time of our method is much less than the other methods; and it can work on big/medium data (i.e., the data having more than nearly 250 genes). Fig. 11 presents the comparison of the number of significant non-redundant itemsets among our proposed method (i.e., *StatBicRM*) and the other methods for details. S3 Text shows the comparative study about the number of evolved rules as well as total elapsed time among our proposed rule mining method and the other rule mining methods for the two artificial binary datasets (i.e., *ArDS*5 and *ArDS*6).

### Integrative analysis of Gene Expression dataset and Methylation dataset

For integrative analysis of gene expression dataset and methylation dataset, we have to consider only matched samples and matched genes from both the datasets (having same dataset Reference ID). Dataset 2 (NCBI Ref. ID:- GSE31699, gene expression dataset) and Dataset 3 (NCBI Ref. ID:- GSE31699, methylation dataset) have 13072 common genes (i.e., combined dataset). For integrative analysis of the combined dataset, at first we have identified 16 matched samples from the combined dataset. Thereafter, we have used the normality test on the matched expression dataset as well as matched methylation dataset individually. For the matched expression dataset, we have identified that 10236 of the matched genes are normally distributed, and rest of them (i.e., 2836 genes) are not normally distributed. For the matched methylation dataset, we have found 8173 genes which are following normal distribution, and remaining 4899 genes that do not following normal distribution. Then, the four parametric tests are applied on the normally distributed genes, and the four non-parametric tests are applied on the non-normally distributed genes, both at 0.05 p-value threshold for the matched expression dataset as well as the matched methylation dataset. We have found 54 common up-regulated genes and 82 common down-regulated genes from the parametric tests, and 86 common up-regulated genes and 70 common down-regulated genes from the non-parametric tests for the expression dataset (viz., ∣*UPDESET*_*N*_∣ = 54, ∣*UPDESET*_*NN*_∣ = 86, ∣*DOWNDESET*_*N*_∣ = 82 and ∣*DOWNDESET*_*NN*_∣ = 70). Thereafter, we merge the common up-regulated and common down-regulated genes collected from the results of the parametric and nonparametric tests for the matched expression dataset (viz., ∣*TOTALDESET*_(*N*+*NN*)_∣ = 292). Similar statistical analyses are performed for the matched methylation dataset (viz., ∣*HYPERDMSET*_*N*_∣ = 89, ∣*HYPERDMSET*_*NN*_∣ = 174, ∣*HYPODMSET*_*N*_∣ = 95, ∣*HYPODMSET*_*NN*_∣ = 165, and ∣*TOTALDMSET*_(*N*+*NN*)_∣ = 523). Thereafter, our proposed method is applied on the *TOTALDESET*_(*N*+*NN*)_ genes as well as the *TOTALDMSET*_(*N*+*NN*)_ genes, individually (see S4 Text).

Furthermore, we have concentrated on the internal relationship between the gene expression and methylation. As we know that the gene expression is inversely proportional to the methylation, therefore inversely correlated genes make sense to highlight the effect of methylation (i.e., epigenetic effect) on the expression level. For the combined dataset, six common genes have been identified which are up-regulated as well as hypo-methylated. These genes are SEMA7A, FSD1, TUBB3, GLIS1, TDO2 and SHOX2. Subsequently, nine common genes are also detected that are both down-regulated and hyper-methylated. These genes are HOXB8, SLC25A18, CALCRL, TMEM71, C1orf115, EDG1, CCDC68, NUAK1 and CMTM8. These two types of genes are important for highlighting the effect of methylation (i.e., epigenetic effect) on the gene expression. However, the supplementary materials are available at: https://www.dropbox.com/sh/gsvgty85jdlp2b6/13fOZxJV8n.

## Conclusion

In this article, we have proposed a computational rule mining framework to determine special type of association rules and potential biomarkers using integrated approaches of statistical and the BiMax biclustering techniques from the gene expression as well as methylation data. At first, different statistical techniques (viz., removal of genes having low variance, normalization, normality test, different parametric and non-parametric tests) are utilized on the the whole dataset to obtain proper non-redundant/high-significant subset of differentially expressed/methylated genes. The resulting subset of genes are used in next step. Thereafter, the data is discretized and post-discretized, consecutively. The biclustering technique is then utilized to determine *MFCHOIs*. Thereafter, the special rules are generated from the *MFCHOIs*. Our proposed rule mining method performs much better than the state-of-the-art rule mining algorithms as it generates *MFCHOIs* instead of *FIs*. Therefore, it saves running time, and it can able to work on the big dataset. Pathway and *GO* analyses have been performed on the genes of the evolved rules by David software. Occurrence of each gene in the evolved rules of each class-label is determined for identifying the potential biomarkers. Furthermore, we have also made classification the data to know how much the evolved rules are able to describe accurately the remaining test (unknown) data. Subsequently, we have also compared the average classification accuracy, and other related factors of our proposed method with the other existing rule-based classifiers. Statistical significance tests are also utilized for checking the statistical relevance of the comparisons. Here, each of the other rule mining methods or rule-based classifiers is also starting with the same post-discretized data-matrix. At the end, we have also performed the integrated analysis of the gene expression data and the methylation data for highlighting the epigenetic effect (viz., the effect of methylation) on the gene expression level.

## Supporting Information

S1 Text Brief descriptions about the used rule-interestingness measures. (PDF)

S2 Text Top 15 rules of treated/experimental and control class labels from the results of average ranking for the three real datasets. (PDF)

S3 Text Comparison among our proposed rule mining method and the other existing rule mining methods for the two artificial datasets. (PDF)

S4 Text Integrative analysis of Gene Expression dataset and Methylation dataset. (PDF)

S1 File Significant KEGG pathways, GO:BPs, GO:CCs and GO:MFs of the genes of the evolved rules for Dataset 1. (XLS)

S2 File Significant KEGG pathways, GO:BPs, GO:CCs and GO:MFs of the genes of the evolved rules for Dataset 2. (XLS)

S3 File Significant KEGG pathways, GO:BPs, GO:CCs and GO:MFs of the genes of the evolved rules for Dataset 3. (XLS)
