# Supplementary material for: Analyzing Large Gene Expression and Methylation Data Profiles Using StatBicRM: Statistical Biclustering-Based Rule Mining
Source: PLoS One. 2015 Apr 1;10(4):e0119448. doi: 10.1371/journal.pone.0119448 (PMC4382191; doi:10.1371/journal.pone.0119448)
Supplement: S1 Text — (PDF) [file pone.0119448.s001.pdf]

TABLE I: A real-life example of transaction data matrix

**Bread**	**Butter**	**Milk**
1	1	0
0	1	0
0	1	1
1	1	1
1	0	1

TABLE II: 2 × 2 Contingency for the rule $A \rightarrow C$

	**C**	**¬C**	
A	n(AC)	n(A$\neg C$)	n(A)
$\neg A$	n($\neg AC$)	n($\neg A C$)	n($\neg A$)
	n(C)	n($\neg C$)	N

I. Rule-interestingness measures

We have used 24 rule-interestingness measures. Among these, 23 are existing. These 23 measures are presented in Table III.

Suppose, $IM = \{i_1, i_2, ..., i_p\}$ denotes an itemset (where p denotes the number of items in the itemset), and N be the total number of transactions. Here, each transaction (i.e., t) is a set of items by which $t \subseteq IM$ must occur. Let, $A \rightarrow C$ be an association rule (by Agrawal and Srikant, 1994), where $A, C \subseteq IM$ and $A \cap C = \phi$. Here, A and C denote its antecedent (i.e., set of items in left hand side of a rule) and consequent (i.e., set of items in right hand side of a rule), respectively. The support of the itemset is defined as number of transactions in which all items of it appear. The confidence of the rule is defined as ratio of support of the itemset to the support of its antecedent. The support and confidence were the original rule-interestingness measures which were proposed for association rules (by Agrawal and Srikant, 1994). There are two major interestingness criteria for any rule. These are generality and reliability. Coverage or support indicates the generality of the rule, where added value or confidence or lift presents the reliability of the rule. Let, TD is the transaction data matrix presented in Table I. There are five transactions (or, rows) and three items (viz., bread, butter and milk). In Table I, 1 stand for an item which occurs in the transaction, and 0 signifies that the item does not occur in the transaction. Suppose, an association rule is: $Bread \rightarrow Butter$ that is mined from TD. The support of the rule will be 0.40 as bread and butter occurs together in two out of five transactions. The confidence of the rule will be 0.67 as butter occurs in two out of the three transactions which contain bread.

Table II shows a 2 × 2 contingency table which have the frequency counts of satisfying A, $\neg A$, C and $\neg C$, pairwise in N number of transactions (where $n(AC)$ denotes the number of transactions satisfying both A and C). Here, the probability of A is presented by $P(A) = \frac{n(A)}{N}$, and the conditional probability of C is denoted by $P(C|A) = \frac{P(AC)}{P(A)}$ (where, A is given). These measures are taken from various sources like statistics (ø-correlation coefficient and Yules Q), and information retrieval (sensitivity and accuracy).

ø-correlation coefficient (Agresti 1990) is closely related to the χ^2 statistic as $\phi^2 = \chi^2/N$. As it depends on the size of the database, thus it is utilized as a rule-interestingness measure.

Tan et al. [2000] proposed IS measure (viz., $IS = \sqrt{I \times support}$, where $I = \frac{P(AB)}{P(A)P(B)}$), which is the geometric mean of interest factor (I) and support measures. It is derived from the ø-correlation coefficient measure. In case of pairs of items, the IS measure is equivalent to the cosine measure which is useful specially for similarity measure for vector-space models.

Interest factor is a rule-interestingness measure which is extensively utilized in data mining for calculating deviation from statistical independence. This measure is sensitive to the support of the items. The variants of it include added value (Sahar et al. 1999) and leverage-2 (Piatetsky-Shapiro 1991) measures. A weighted relative accuracy (i.e., $WRAcc = P(A)(P(C|A) - P(C))$) was proposed by Lavrac et al. 1999. It merges the coverage and the added value. This measure is similar to leverage-2 measure (i.e., $P(AC) - P(A)P(C)$). Other rule-interestingness measures which are related to these two criteria, include Jaccard (Tan et al. 2002), two-way support (Yao and Zhong 1999) and Klosgen (Klosgen 1996). These three measures merge either support (i.e., P(AC)) or coverage (i.e., P(A)) with a correlation factor of either lift (viz., $P(C|A)/P(C)$) or ($P(C|A) - P(C)$). The Jaccard measure is utilized specially for information retrieval to measure the similarity between documents. The Klosgen measure is useful for Explora knowledge discovery system.

Odds ratio is a rule-interestingness measure which comprises the odds for acquiring the different outcomes of a variable. The ranges of the odds ratio value are from 0 (for perfect negative correlation) to N (for perfect positive correlation). Yule's Q (Yule 1900) is a normalized variant of the odds ratio. Yule's Q has the value ranging from -1 to +1.

In 1999, Bayardo and Agrawal defined the relationships among confidence, support and other rule-interestingness measures from a different point of view. They proposed a definition of a partial ordered relation depending confidence and support. It is stated as: "in case of rules 'R1' and 'R2', if $confidence(R2) \geq confidence(R1)$ and $support(R2) \geq support(R1)$ exist, then $R2 \geq_{sc} R1$ (where sc denotes sc-optimal rule). The rule (i.e., R) in the upper border in which no other rule (i.e., R') can exist by which $R' \geq_{sc} R$ satisfies, is stated as a sc-optimal rule. A rule should be sc-optimal if any of its measures is monotone

TABLE III: The formulas of rule-interestingness measures.

#	Measure	Formula
1	**Support**	P(AC)
2	**Confidence**	max $\{P(C\|A), P(A\|C)\}$
3	**Coverage**	P(A)
4	**Prevalence**	P(C)
5	**Sensitivity (or, recall)**	$P(A\|C)$
6	**Specificity**	$P(\neg C\|\neg A)$
7	**Accuracy**	$P(AC) + P(\neg A \neg C)$
8	**Lift (or, interest)**	$P(C\|A)/P(C)$ or $P(AC)/P(A)P(C)$
9	**Leverage**	$P(C\|A) - P(A)P(C)$
10	**Added value**	max$\{P(C\|A) - P(C), P(A\|C) - P(A)\}$
11	**Relative risk**	$\frac{P(C\|A)}{P(C\|\neg A)}$
12	**Jaccard**	$P(AC)/(P(A) + P(C) - P(AC))$
13	**Yule's Q**	$\frac{P(AC)P(\neg A \neg C) - P(A \neg C)P(\neg AC))}{P(AC)P(\neg A \neg C) + P(A \neg C)P(\neg AC))}$
14	**Klosgen**	$\sqrt{P(AC)} max\{P(C\|A) - P(C), P(A\|C) - P(A)\}$
15	**Laplace correction**	$\frac{N(AC)+1}{N(A)+2}$
16	**Gini index**	$P(A) * \{P(C\|A)^2 + P(\neg C\|A)^2\} + P(\neg A) * \{P(C\|\neg A)^2 + P(\neg C\|\neg A)^2\} - P(C)^2 - P(\neg C)^2$
17	**Two-way support**	$P(AC) * log_2 \frac{P(AC)}{P(A)P(C)}$
18	**Linear correlation coefficient (or, ϕ-coefficient)**	$\frac{P(A,C) - P(A)P(C)}{\sqrt{P(A)P(C)(1-P(A))(1-P(C))}}$
19	**Cosine**	$\frac{P(AC)}{\sqrt{P(A)P(C)}}$
20	**Least contradiction**	$\frac{P(AC) - P(A \neg C)}{P(C)}$
21	**Zhang**	$\frac{P(AC) - P(A)P(C)}{max\{P(AC)P(\neg C), P(C)P(A \neg C)\}}$
22	**Leverage 2 (or, Piatetsky-Shapiro)**	$P(AC) - P(A)P(C)$
23	**Kappa**	$\frac{P(AC) + P(\neg A \neg C) - P(A)P(C) - P(\neg A)P(\neg C))}{1 - P(A)P(C) - P(\neg A)P(\neg C)}$

in both confidence and support. E.g., the Laplace correction measure (viz., $\frac{n(AC)+1}{n(A)+2}$) (by Clark and Boswell 1991) can be rewritten as $\frac{N \times support(A \to C)+1}{N \times (support(A \to C)/confidence(A \to C))+2}$; i.e., it is a function of $confidence(A \to C)$ and $support(A \to C)$. It can be shown easily that the Laplace correction measure is monotone in both confidence and support. This property is utilized if the user keeps his attention only in a single most interesting rule, as it is only necessary to verify the sc-optimal ruleset that has fewer rules than the whole ruleset.
