# Supplementary material for: Analyzing Large Gene Expression and Methylation Data Profiles Using StatBicRM: Statistical Biclustering-Based Rule Mining
Source: PLoS One. 2015 Apr 1;10(4):e0119448. doi: 10.1371/journal.pone.0119448 (PMC4382191; doi:10.1371/journal.pone.0119448)
Supplement: S2 Text — (PDF) [file pone.0119448.s002.pdf]

TABLE I: Top 15 association rules of treated and control class labels from the results of average ranking for (a) Dataset 1, (b) Dataset 2 and (c) Dataset 3; here '+' denotes up-regulation and '-' denotes down-regulation for Dataset 1 and Dataset 2, where '+' denotes hyper-methylation and '-' denotes hypo-methylation for Dataset 3.

(a)

{BNC1+, KRT13+, SERPINB13+, SPRR1A+, ⇒ class=SCC}
{BNC1+, KRT13+, SERPINB13+, CMTM8- ⇒ class=SCC}
{BNC1+, SERPINB13+ ⇒ class=SCC}
{SPRR1A+, ATP11A- ⇒ class=SCC}
{SPRR1A+, ATP11A-, SULT1A2- ⇒ class=SCC}
{ATP11B+, BNC1+, CHST7+, KRT16+, SERPINB13+ ⇒ class=SCC}
{SERPINB13+, ABHD14A-, CMTM8- ⇒ class=SCC}
{SERPINB13+, COL7A1+, CMTM8- ⇒ class=SCC}
{ACAP2+, SERPINB13+, ⇒ class=SCC}
{KRT14+, ATP11A-, SHROOM3- ⇒ class=SCC}
{ATP11A-, CMTM8-, SHROOM3- ⇒ class=SCC}
{SPRR1A+, SPSB2- ⇒ class=SCC}
{SERPINB13+, CMTM8- ⇒ class=SCC}
{SPRR1A+, EPS8L2- ⇒ class=SCC}
{BNC1+, KRT13+, SERPINB13+, ATP11A-, SHROOM3- ⇒ class=SCC}

(b)

{JAG1-, PECAM1- ⇒ class=tumor}
{FLJ10986+, CDC34+ ⇒ class=tumor}
{MCM4+, KRT17+ ⇒ class=tumor}
{ZNF217+, ACSL5+ ⇒ class=normal}
{TIGD7+, CDC34+ ⇒ class=tumor}
{JAG1-, FBXO33-, NUAK1-, PSCD1- ⇒ class=tumor}
{PRL+, TDO2+, EGFL6+ ⇒ class=tumor}
{JAG1-, PPP2R2B-, SASH1- ⇒ class=tumor}
{CDC34+, PRKCH- ⇒ class=tumor}
{PRL+, NUAK1- ⇒ class=tumor}
{PRL+, TRPC6+ ⇒ class=tumor}
{AOX1+, GSTA4- ⇒ class=normal}
{MCM4+, PRL+ ⇒ class=tumor}
{PRRG1+, ACSL5+ ⇒ class=normal}
{FBXO33-, PPP2R2B- ⇒ class=tumor}

(c)

{KLF11+, STEAP4+ ⇒ class=tumor}
{ZMYND15+, C11orf38- ⇒ class=tumor}
{SLC15A3+, APOB+ ⇒ class=tumor}
{ACTN2+, CALCRL+ ⇒ class=tumor}
{STEAP4+, DLEC1+, APOB+ ⇒ class=tumor}
{CST9L+, S100A16+ ⇒ class=normal}
{KLF11+, IL20RA+ ⇒ class=tumor}
{C1orf115+, ZMYND15+ ⇒ class=tumor}
{INHBE-, LYZL2- ⇒ class=normal}
{DLEC1+, APOB+ ⇒ class=tumor}
{DLEC1+, TRPM2- ⇒ class=tumor}
{MGC16291-, SMPD2- ⇒ class=tumor}
{TRPM2-, SMPD2- ⇒ class=tumor}
{CST9L+, NODAL- ⇒ class=normal}
{C11orf38-, TEX101- ⇒ class=tumor}
