# Supplementary material for: Analyzing Large Gene Expression and Methylation Data Profiles Using StatBicRM: Statistical Biclustering-Based Rule Mining
Source: PLoS One. 2015 Apr 1;10(4):e0119448. doi: 10.1371/journal.pone.0119448 (PMC4382191; doi:10.1371/journal.pone.0119448)
Supplement: S3 Text — (PDF) [file pone.0119448.s003.pdf]

Here, we compare the number of evolved rules among our proposed rule mining method and the other existing rule mining methods for the two artificial binary datasets (viz., $ArDS5$ and $ArDS6$) in Table 1 and Table 2, respectively. Furthermore, we have compared the elapsed time for extracting the evolved rules among our proposed rule mining method and the other existing rule mining methods for the two artificial datasets (viz., $ArDS5$ and $ArDS6$) in Table 3 and Table 4, respectively. Please note that in our proposed method (i.e., *StatBicRM*) only, each significant non-redundant itemset (viz., maximal frequent closed homogeneous itemset) generates a single special rule, where the in the other methods, each significant itemset (viz., frequent itemset) generates many association rules.

Table 1. Comparison of the number of evolved rules among our proposed rule mining method and the other rule mining methods for the artificial binary dataset (viz., $ArDS5$) at different minimum support and a fixed minimum confidence threshold. Here, $minSp$ means minimum support threshold, and $minCf$ refers to minimum confidence threshold. For only the case of our proposed rule mining method (i.e., *StatBicRM*), no $minCf$ is needed as here each evolved significant itemset denotes an individual rule.

		StatBicRM	**H-mine**	**Tao et al.**	**Eclat**	**AprioriTid**	**Apriori**
**minSp**	**minCf**	**#Rule**	**#Rule**	**#Rule**	**#Rule**	**#Rule**	**#Rule**
0.050	0.5	87	1.32 lakh	1.56 lakh	1.58 lakh	1.58 lakh	1.59 lakh
0.075	0.5	78	1.20 lakh	1.41 lakh	1.42 lakh	1.45 lakh	1.48 lakh
0.100	0.5	58	1.11 lakh	1.27 lakh	1.28 lakh	1.32 lakh	1.36 lakh
0.125	0.5	47	1.05 lakh	1.18 lakh	1.19 lakh	1.21 lakh	1.23 lakh
0.150	0.5	35	93,256	1.09 lakh	1.08 lakh	1.11 lakh	1.14 lakh
0.175	0.5	29	82,528	1.05 lakh	1.06 lakh	1.08 lakh	1.09 lakh
0.200	0.5	24	52,141	82,957	84,578	92,843	95,014

Table 2. Comparison of the number of evolved rules among our proposed rule mining method and the other rule mining methods for the artificial binary dataset (viz., $ArDS6$) at different minimum support and a fixed minimum confidence threshold. Here, $minSp$ means minimum support threshold, and $minCf$ refers to minimum confidence threshold. For the case of only $StatBicRM$, no $minCf$ is needed as here each evolved significant itemset denotes an individual rule.

		StatBicRM	**H-mine**	**Tao et al.**	**Eclat**	**AprioriTid**	**Apriori**
**minSp**	**minCf**	**#Rule**	**#Rule**	**#Rule**	**#Rule**	**#Rule**	**#Rule**
0.050	0.5	168	3.28 lakh	3.52 lakh	3.56 lakh	3.63 lakh	3.68 lakh
0.075	0.5	126	3.25 lakh	3.38 lakh	3.45 lakh	3.53 lakh	3.65 lakh
0.100	0.5	102	3.09 lakh	3.23 lakh	3.32 lakh	3.45 lakh	3.51 lakh
0.125	0.5	83	3.08 lakh	3.15 lakh	3.21 lakh	3.36 lakh	3.43 lakh
0.150	0.5	68	2.95 lakh	3.12 lakh	3.16 lakh	3.28 lakh	3.32 lakh
0.175	0.5	56	2.80 lakh	3.01 lakh	3.08 lakh	3.15 lakh	3.21 lakh
0.200	0.5	38	2.57 lakh	2.85 lakh	2.93 lakh	3.02 lakh	3.06 lakh

Table 3. Comparison of the elapsed time for extracting the evolved rules among our proposed rule mining method and the other rule mining methods for the artificial binary dataset (viz., $ArDS5$) at different minimum support and a fixed minimum confidence threshold. Here, $minSp$ means minimum support threshold, and $minCf$ refers to minimum confidence threshold. For the case of only $StatBicRM$, no $minCf$ is needed as here each evolved significant itemset denotes an individual rule.

		StatBicRM	**H-mine**	**Tao et al.**	**Eclat**	**AprioriTid**	**Apriori**
**minSp**	**minCf**	**Time (Sec.)**	**Time (Sec.)**	**Time (Sec.)**	**Time (Sec.)**	**Time (Sec.)**	**Time (Sec.)**
0.050	0.5	2.23	276.50	365.01	366.18	366.34	367.54
0.075	0.5	2.12	206.28	319.36	320.24	323.57	325.72
0.100	0.5	2.08	158.38	248.76	256.42	286.26	290.38
0.125	0.5	1.53	148.21	203.45	206.21	208.28	215.69
0.150	0.5	1.32	136.11	155.51	152.16	159.18	159.93
0.175	0.5	1.21	115.24	149.41	150.62	152.34	155.66
0.200	0.5	1.02	102.63	119.41	125.24	131.73	138.08

Table 4. Comparison of the elapsed time for extracting the evolved rules among our proposed rule mining method and the other rule mining methods for the artificial binary dataset (viz., $ArDS6$) at different minimum support and a fixed minimum confidence threshold. Here, $minSp$ means minimum support threshold, and $minCf$ refers to minimum confidence threshold. For the case of only $StatBicRM$, no $minCf$ is needed as here each evolved significant itemset denotes an individual rule.

		StatBicRM	**H-mine**	**Tao et al.**	**Eclat**	**AprioriTid**	**Apriori**
**minSp**	**minCf**	**Time (Sec.)**	**Time (minute)**	**Time (minute)**	**Time (minute)**	**Time (minute)**	**Time (minute)**
0.050	0.5	2.54	63.96	69.28	72.02	76.71	82.36
0.075	0.5	2.48	63.86	66.03	67.46	69.93	78.16
0.100	0.5	2.46	55.53	63.72	64.31	67.72	69.25
0.125	0.5	2.42	55.25	60.14	63.28	65.94	67.14
0.150	0.5	2.37	50.14	58.76	60.97	64.06	65.22
0.175	0.5	2.02	43.35	52.26	57.24	60.25	63.62
0.200	0.5	1.54	40.97	45.34	48.52	52.34	55.12
