# Supplementary material for: Analyzing Large Gene Expression and Methylation Data Profiles Using StatBicRM: Statistical Biclustering-Based Rule Mining
Source: PLoS One. 2015 Apr 1;10(4):e0119448. doi: 10.1371/journal.pone.0119448 (PMC4382191; doi:10.1371/journal.pone.0119448)
Supplement: S4 Text — (PDF) [file pone.0119448.s004.pdf]

Integrative analysis of Gene Expression dataset and Methylation dataset

For integrative analysis of gene expression dataset and methylation dataset, we have to consider only matched samples and matched genes from both the datasets (having same dataset Reference ID). Dataset 2 (NCBI Ref. ID:- GSE31699, gene expression dataset) and Dataset 3 (NCBI Ref. ID:- GSE31699, methylation dataset) have 13072 common genes (i.e., combined dataset). For integrative analysis of the combined dataset, at first we have identified 16 matched samples from the combined dataset. Thereafter, we have used the normality test on the matched expression dataset as well as matched methylation dataset individually. For the matched expression dataset, we have identified that 10236 of the matched genes are normally distributed, and rest of them (i.e., 2836 genes) are not normally distributed. For the matched methylation dataset, we have found 8173 genes which are following normal distribution, and remaining 4899 genes that do not following normal distribution. Then, the four parametric tests are applied on the normally distributed genes, and the four non-parametric tests are applied on the non-normally distributed genes, both at 0.05 p-value threshold for the matched expression dataset as well as the matched methylation dataset. We have found 54 common up-regulated genes and 82 common down-regulated genes from the parametric tests, and 86 common up-regulated genes and 70 common down-regulated genes from the non-parametric tests for the expression dataset (viz., $|UPDESET_N| = 54$, $|UPDESET_{NN}| = 86$, $|DOWNDESET_N| = 82$ and $|DOWNDESET_{NN}| = 70$). Thereafter, we merge the common up-regulated and common down-regulated genes collected from the results of the parametric and nonparametric tests for the matched expression dataset (viz., $|TOTALDESET_{(N+NN)}| = 292$). Similar statistical analyses are performed for the matched methylation dataset (viz., $|HYPERDMSET_N| = 89$, $|HYPERDMSET_{NN}| = 174$, $|HYPODMSET_N| = 95$, $|HYPODMSET_{NN}| = 165$, and $|TOTALDMSET_{(N+NN)}| = 523$). Thereafter, our proposed method is applied on the $TOTALDESET_{(N+NN)}$ genes as well as the $TOTALDMSET_{(N+NN)}$ genes, individually. Table 2 presents the comparative performance of our proposed classification method with the existing rule-based classifiers for the matched expression dataset (i.e., using $TOTALDESET_{(N+NN)}$ genes), where Table 1 shows the comparative performance of our proposed classification method with the existing rule-based classifiers for the matched methylation dataset (i.e., using $TOTALDMSET_{(N+NN)}$ genes).

Table 1. Comparative performance analysis of the rule-based classifiers on the matched methylation dataset of the combined dataset, respectively (at 4-fold CVs repeating for 10 times); where bold font denotes the highest value for each column.

Rule-based classifier	Average sensitivity[%] (s.d.)	Average specificity[%] (s.d.)	Average accuracy[%] (s.d.)	Average MCC (s.d.)
**Proposed**	**90.89** (3.05)	86.32 (2.16)	**88.86** (2.58)	**0.77** (0.043)
**ConjunctiveRule**	70.67 (2.87)	91.03 (6.53)	80.56 (2.27)	0.63 (0.068)
**DecisionTable**	84.15 (3.76)	82.96 (4.47)	83.36 (0.93)	0.68 (0.028)
**JRip**	76.23 (2.48)	92.42 (2.97)	85.35 (1.69)	0.70 (0.026)
**OneR**	76.85 (7.79)	88.06 (4.95)	82.36 (1.57)	0.66 (0.042)
**PART**	76.95 (12.89)	**94.96** (0.51)	85.67 (6.97)	0.72 (0.126)
**Ridor**	83.05 (4.23)	80.56 (8.72)	81.76 (2.84)	0.64 (0.042)

Furthermore, we have concentrated on the internal relationship between the gene expression and methylation. As we know that the gene expression is inversely proportional to the methylation, therefore inversely correlated genes make sense to highlight the effect of methylation (i.e., epigenetic effect) on

Table 2. Comparative performance analysis of the rule-based classifiers on the matched expression dataset of the combined dataset, respectively (at 4-fold CVs repeating for 10 times); where bold font denotes the highest value for each column.

Rule-based classifier	Average sensitivity[%] (s.d.)	Average specificity[%] (s.d.)	Average accuracy[%] (s.d.)	Average MCC (s.d.)
**Proposed**	**98.13** (3.02)	53.75 (3.23)	**75.94** (1.51)	**0.58** (0.037)
**ConjunctiveRule**	71.88 (8.46)	63.75 (8.23)	67.81 (3.91)	0.36 (0.081)
**DecisionTable**	76.88 (3.02)	62.5 (5.10)	69.69 (1.51)	0.40 (0.027)
**JRip**	70.63 (3.02)	62.5 (5.10)	66.56 (3.92)	0.33 (0.080)
**OneR**	73.13 (3.02)	58.75 (10.70)	65.93 (4.53)	0.33 (0.087)
**PART**	66.25 (6.04)	**64.38** (7.83)	65.31 (2.74)	0.31 (0.057)
**Ridor**	76.88 (3.02)	58.75 (3.23)	67.81 (1.51)	0.37 (0.031)

the expression level. For the combined dataset, six common genes have been identified which are up-regulated as well as hypo-methylated. These genes are SEMA7A, FSD1, TUBB3, GLIS1, TDO2 and SHOX2. Subsequently, nine common genes are also detected that are both down-regulated and hyper-methylated. These genes are HOXB8, SLC25A18, CALCRL, TMEM71, C1orf115, EDG1, CCDC68, NUAK1 and CMTM8. These two types of genes are important for highlighting the effect of methylation (i.e., epigenetic effect) on the gene expression.
